# The spectrum of neutralizing and non-neutralizing anti-FVIII antibodies in a nationwide cohort of 788 persons with hemophilia A

**DOI:** 10.3389/fimmu.2024.1355813

**Published:** 2024-02-22

**Authors:** Ilja Oomen, Marieke Verhagen, Mariarosaria Miranda, Peter Allacher, Erik A. M. Beckers, Nicole M. A. Blijlevens, Johanna G. van der Bom, Michiel Coppens, Mariëtte Driessens, Jeroen C. J. Eikenboom, Karin Fijnvandraat, Shermarke Hassan, Waander L. van Heerde, H. Louise Hooimeijer, Joop H. Jansen, Paul Kaijen, Frank W. G. Leebeek, Daniëlle Meijer, Helmut Paul, Sanna R. Rijpma, Frits R. Rosendaal, Cees Smit, Lize F. D. van Vulpen, Jan Voorberg, Saskia E. M. Schols, Samantha C. Gouw

**Affiliations:** ^1^ Department of Pediatric Hematology, Amsterdam University Medical Center (UMC) Location University of Amsterdam, Amsterdam, Netherlands; ^2^ Department of Molecular Hematology, Sanquin Research, Amsterdam, Netherlands; ^3^ Department of Hematology, Radboud University Medical Center, Nijmegen, Netherlands; ^4^ Hemophilia Treatment Center Nijmegen-Eindhoven-Maastricht, Nijmegen, Netherlands; ^5^ Laboratory of Hematology, Department of Laboratory Medicine, Radboud University Medical Center, Nijmegen, Netherlands; ^6^ Institute Krems Bioanalytics, International Management Center (IMC) University of Applied Sciences Krems, Krems, Austria; ^7^ Division of Hematology, Department of Internal Medicine, Maastricht University Medical Center, Maastricht University, Maastricht, Netherlands; ^8^ Department of Clinical Epidemiology, Leiden University Medical Center, Leiden, Netherlands; ^9^ Department of Vascular Medicine, Amsterdam Cardiovascular Sciences, Amsterdam University Medical Centers, Location University of Amsterdam, Amsterdam, Netherlands; ^10^ Dutch Society of Hemophilia Patients, Leiden, Netherlands; ^11^ Division of Thrombosis and Hemostasis, Department of Internal Medicine, Leiden University Medical Center, Leiden University, Leiden, Netherlands; ^12^ Infectious Diseases Data Observatory, Center for Tropical Medicine and Global Health, University of Oxford, Oxford, United Kingdom; ^13^ Enzyre BV, Nijmegen, Netherlands; ^14^ Division of Hematology/Oncology, Department of Pediatrics, University Medical Center Groningen, University of Groningen, Groningen, Netherlands; ^15^ Department of Hematology, Erasmus University Medical Center, Rotterdam, Netherlands; ^16^ Center for Benign Hematology, Thrombosis and Haemostasis, Van Creveldkliniek, University Medical Center Utrecht, University Utrecht, Utrecht, Netherlands

**Keywords:** antibody, ELISA, factor VIII, hemophilia A, immunoglobulin A, immunoglobulin G, immunoglobulin M

## Abstract

**Objectives:**

Anti-factor VIII (FVIII) antibodies have been reported to exhibit both neutralizing and non-neutralizing characteristics. This is the first study investigating the full spectrum of FVIII-specific antibodies, including non-neutralizing antibodies, very-low titer inhibitors, and inhibitors, in a large nationwide population of persons with hemophilia A of all severities.

**Methods:**

All persons with hemophilia A (mild (FVIII > 5–40 IU/dL)/moderate [FVIII 1–5 IU/dL)/severe (FVIII < 1 IU/dL)] with an available plasma sample who participated in the *sixth Hemophilia in the Netherlands* study between 2018 and 2019 were included. The presence of anti-FVIII antibodies of the immunoglobulin A, M, and G isotypes and IgG subclasses, along with antibody titer levels, were assessed using direct-binding ELISAs. FVIII specificity was assessed using a competition-based ELISA approach. The inhibitor status was determined using the Nijmegen ultra-sensitive Bethesda assay (NusBA) and the Nijmegen Bethesda assay (NBA).

**Results:**

In total, 788 persons with hemophilia A (336 (42.6%) mild, 123 (15.6%) moderate, 329 (41.8%) severe hemophilia) were included. The median age was 45 years (IQR 24–60), and the majority (50.9%) had over 150 exposure days to FVIII concentrates. Within our population, 144 (18.3%) individuals had non-neutralizing FVIII-specific antibodies, 10 (1.3%) had very low-titer inhibitors (NusBA positive; NBA negative), and 13 (1.6%) had inhibitors (both NusBA and NBA positive). IgG1 was the most abundant FVIII-specific antibody subclass, and the highest titer levels were found for IgG4. In individuals without a reported history of inhibitor development, no clear differences were observed in antibody patterns between those who were minimally or highly exposed to FVIII concentrates. IgG4 subclass antibodies were only observed in persons with a reported history of FVIII inhibitor or in those with a currently detected (very low-titer) inhibitor.

**Conclusion:**

In this cross-sectional study, we identified non-neutralizing antibodies in a relatively large proportion of persons with hemophilia A. In contrast, in our population, consisting of persons highly exposed to FVIII concentrates, (very low-titer) inhibitors were detected only in a small proportion of persons, reflecting a well-tolerized population. Hence, our findings suggest that only a small subpopulation of non-neutralizing FVIII-specific antibodies is associated with clinically relevant inhibitors.

## Introduction

Hemophilia A is an X-linked inherited bleeding disorder in which functional coagulation factor VIII (FVIII) is deficient. Disease severity is defined by the residual FVIII activity level (severe < 1 IU/dL, moderate 1–5 IU/dL, and mild > 5–40 IU/dL), which generally correlates with the clinical bleeding phenotype (1). Typically, persons with severe hemophilia A have an increased risk of spontaneous bleeding, especially in joints and muscles (1). Prophylactic therapy with clotting factor concentrates and FVIII mimicking agents (nonfactor concentrates) are being used to prevent bleeding and preserve musculoskeletal health (1).

The development of neutralizing anti-FVIII antibodies, called inhibitors, is a major complication of hemophilia care. These inhibitors bind to functional sites on FVIII thereby impairing the pro-coagulant efficacy of FVIII concentrates (2). Consequently, inhibitors cause substantial morbidity and increase mortality due to severe bleeding episodes (3). The only proven method to eradicate these inhibitors is immune tolerance induction therapy, consisting of frequently high doses of FVIII concentrates (1). In severe hemophilia A, approximately 30% develop inhibitors, typically within the first 50 days of exposure to FVIII concentrates (2, 4, 5). In mild to moderate-severe hemophilia A, the cumulative incidence of FVIII inhibitors is around 5% to 10% (6). The gold standard method for inhibitor detection is the Bethesda Assay or Nijmegen-modified Bethesda Assay (NBA), with a NBA titer of ≥ 0.6 Bethesda Units (BU)/mL defined as positive (7–9). In these functional assays, inhibitor titers are defined based on the extent of inhibition of FVIII activity in healthy donor plasma by anti-FVIII antibodies present in patient plasma (8, 9). Recently, the Nijmegen ultra-sensitive Bethesda Assay (NusBA) has been developed, allowing for the detection of very low-titer inhibitors down to 0.10 Nijmegen ultra-sensitive Bethesda Units (NusBU)/mL (10).

In literature, also non-neutralizing FVIII-specific antibodies (NNA) have been described, which escape detection in the NBA (11–14). The reported prevalence of these NNA varies widely, caused by the considerable heterogeneity in study design, study population, and type of assays used. Abdi and colleagues reported a pooled NNA prevalence of 25% (95% CI, 16%–38%) in high-quality studies (15). To date, no gold standard method exists to detect these types of antibodies (15). Additionally, large representative population studies investigating the prevalence of NNA are lacking.

Although the clinical significance of NNA is not yet fully understood, NNA could help us further understand the immune response toward exogenous FVIII concentrates. NNA have been reported to be primarily directed toward nonfunctional epitopes of the FVIII protein. However, they can also bind with lower affinity to functional epitopes, which does not lead to neutralization of the FVIII activity (16). Moreover, the presence of NNA may be associated with inhibitor development. The *Survey of Inhibitors in Plasma-Product Exposed Toddlers* (SIPPET) study and the *Prospective Hemophilia Inhibitor Previously Untreated Patients* (*PUP*) *Study* (HIPS) demonstrated that the presence of NNA was associated with an increased risk of inhibitor development in previously untreated persons with hemophilia A (17, 18).

Here, we investigated the full spectrum of neutralizing and non-neutralizing FVIII-specific antibodies in the *sixth Hemophilia in the Netherlands* population of persons with hemophilia A of all severities.

## Materials and methods

### Study design and population

All adult and pediatric persons with mild (FVIII > 5–40 IU/dL), moderate (FVIII 1–5 IU/dL), and severe (FVIII < 1 IU/dL) hemophilia A, with an available plasma sample, were recruited from the *sixth Hemophilia in the Netherlands* study.

The *sixth Hemophilia in the Netherlands* study is a nationwide cross-sectional study that included persons with congenital hemophilia A or B, who were registered at one of the six Dutch Hemophilia Treatment Centers between June 2018 and July 2019. The study was approved by the Medical Ethical Committee of Leiden University Medical Center (registration number NL59144.058.17). All participants and/or parents, in the case of minors, gave written informed consent. Additional information on the *sixth Hemophilia in the Netherlands* study has been published previously (19).

### Outcomes and definition of FVIII-specific antibodies

The primary outcome was the prevalence of NNA, very low-titer inhibitors, and inhibitors in a hemophilia A population of all severities. The secondary outcome was the characteristics of these antibodies, including immunoglobulin isotype and subclass, antibody titer level, NusBA titer, or NBA titer. FVIII-specific antibodies were defined as antibodies specific for FVIII, identified in a competition-based enzyme-linked immunosorbent assay (ELISA) approach. NNA were defined as FVIII-specific antibodies without neutralizing capacity (both NusBA and NBA negative). Very low-titer inhibitors were defined as FVIII neutralizing antibodies with titers ≥ 0.1 Nijmegen ultra-sensitive Bethesda Units (NusBU/mL), but below the limit of quantitation of the NBA (< 0.6 NBU/mL) (NusBA positive and NBA negative) (7, 10). Inhibitors were defined as FVIII neutralizing antibodies detectable with the NBA (≥ 0.6 NBU/mL) (both NusBA and NBA positive).

### Data collection

Clinical data were collected from medical records. If these medical record data were unavailable, data from self-reported questionnaires were used. For this study, the following clinical characteristics were collected: age, severity of hemophilia, treatment regimen (product type and regimen (on-demand or prophylaxis)), cumulative exposure days to FVIII, inhibitor history, and history of immune tolerance induction treatment.

### Blood sampling and plasma preparation

Blood was drawn in 3.2% sodium citrate tubes during a regular visit to the Hemophilia Treatment Center, following a washout period of at least 3 days after the last FVIII concentrate infusion and a minimum of 7 days after resolution of a bleed, according to a uniform *sixth Hemophilia in the Netherlands* blood collection protocol (**Supplementary Methods M1**). Immediately after collection, blood was processed according to a standardized protocol. Samples were centrifuged two times for 15 min at room temperature at 3,000×*g* to obtain platelet-poor plasma. The plasma samples were subsequently aliquoted into 0.5 mL long-term freeze storage tubes and stored at −80°C. The samples were sent on dry ice to Sanquin Research for the ELISAs and to the Laboratory of Hematology of the Radboud University Medical Center for the NBA and NusBA.

### Laboratory analyses

#### Detection of FVIII-binding antibodies

Maxisorp microtiter plates (Nunc) were coated with 1 μg/mL recombinant human nonmodified full-length FVIII (Advate^®^, Takeda, Vienna, Austria) diluted in 0.05M carbonate–bicarbonate buffer (pH 9.8) and incubated for 16 h ± 2 h at 4°C. Plates were washed with phosphate-buffered saline (pH 7.4, Fresenius Kabi B.V., Zeist, Netherlands) with 0.05% Tween-20 (Sigma-Aldrich, St Louis, U.S.A.) using the ELISA-plate washer (AquaMax 2000). The remaining binding sites were blocked by incubating with a buffer containing 2% bovine serum albumin (BSA) (Hyclone, Logan, Utah, U.S.A.) diluted in 0.4 M sodium chloride phosphate-buffered saline solution (NaCl-PBS) for 1 h ± 10 min at room temperature. Participant samples and positive and negative controls (see Positive and negative controls) were diluted at a dilution of 1:20 in 2% BSA in 0.4 M NaCl-PBS buffer and incubated for 2 h ± 10 min at room temperature. Horseradish peroxidase (HRP)-conjugated anti-human detection antibodies for each human Ig isotype and IgG subclass (**Supplementary Table S1**) were appropriately diluted in 2% BSA in 0.4 M NaCl-PBS buffer, applied, and incubated for 1 h ± 10 min at room temperature (**Supplementary Table S1**). All detection antibodies were confirmed for specificity to their appropriate human Ig (**Supplementary Table S2**). HRP-based color development was assessed using a 3,3′,5,5′-tetramethylbenzidine (TMB)-based solution containing 1 mL of TMB, 2 mL of sodium acetate, and 3 μL of hydrogen peroxide and incubated for 5 min for IgA, IgM, IgG1, IgG3, and IgG4, and for 8.5 min for IgG2. The color development was stopped after applying 100 μL of 1 M sulfuric acid in each well. The optical density (OD) for each sample was assessed using a Microplate Reader (SpectraMax Plus 384) at a wavelength of 450 nm and a 540-nm reference.

In-house-produced monoclonal human antibodies with specificity of the C2 domain of FVIII (EL-14 IgG, EL-14 IgM, and EL-14 IgA), as previously described (20), were used for the standard curves. The FVIII-specific single-chain variable fragment EL-14 recognizing the C2 domain was converted into full-length human IgA, IgM, and IgG of different subclasses (21). Each ELISA contained duplicate standard curves. The EL-14 standard curves had different detection ranges for each IgA, IgM, and IgG subclass, as presented in **Supplementary Figures S1A–E**. A signal over two times in the background was considered to be positive. Each plasma sample was analyzed twice at a dilution of 1:20. If the OD value of both analyses was below the cutoff, the sample was deemed negative. If the OD value of both analyses was equal to or greater than the cutoff, the sample was considered positive and subsequently analyzed for antibody titer level (see Determination of antibody titer level). In case of a discrepancy, a third repetition was performed, which determined the final outcome.

Each ELISA included eight duplicates of the negative control and two duplicates of the positive control (see Positive and negative controls for ELISA).

#### Determination of antibody titer levels

Each plasma sample was analyzed twice. A difference of one dilution step between the duplicate assays was the maximal variation accepted. If the difference between the duplicate assays was one dilution step (e.g., 1:20 (dilution step 1) and 1:40 (dilution step 2)), the highest titer level was reported. In the case of a larger variation, a third repetition was done (**Supplementary Table S3**).

#### Confirmation of FVIII-specificity

Plasma samples and controls (see Positive and negative controls for ELISA) were preincubated with 100 μg/mL recombinant FVIII for 1 h ± 10 min at room temperature. Subsequently, samples and controls were pipetted into wells and incubated for 2 h ± 10 min at room temperature. FVIII specificity was defined as a decline in OD of at least 50% obtained for a specific dilution of patient plasma in the presence of FVIII (**Supplementary Figure S2**).

#### Positive and negative controls for ELISA

The positive controls involved in-house-produced EL-14 FVIII-specific human monoclonal antibodies (20, 21) of each respective IgA, IgM, and IgG subclass spiked into pooled human plasma from 40 healthy donors. A similar pooled human plasma pool was used as a negative control.

#### Nijmegen ultra-sensitive Bethesda Assay

Patient plasma and FVIII deficient pooled plasma (HRF, Inc. Raleigh, NC, USA) were preheated for 1.5 h at 58°C and centrifuged for 5 min at 18,000×*g*, to remove residual FVIII activity that may interfere with the assay. Both patient and reference plasma (FVIII deficient pooled plasma) were mixed with 0.1M Imidazole buffered normal pooled plasma (NNP) (pH 7.4) in a ratio of 9:1 (180:20 μL). These mixtures were incubated for 2 hours at 37°C. Afterward, the remaining FVIII activity level (FVIII:C) was measured with the Biophen FVIII:C Chromogenic assay (Hyphen Biomed, Neuville-Sur-Oise, France) at the STAR Max3 (Diagnostic Stago, Asnières sur Seine, France), according to the manufacturer’s instructions. Residual FVIII activity was defined as the percentage of remaining FVIII:C in the test plasma compared to the FVIII:C in the reference plasma. The previously described formula was used to calculate NusBA titer expressed in NusBU/mL (10). A NusBA titer of ≥0.10 NusBU/mL was defined as positive (10). For samples containing emicizumab, the NusBA was performed using bovine reagents (Biophen FVIII Variant, Hyphen Biomed, Paris, France).

#### Nijmegen Bethesda assay

Patient and reference plasma were prepared as described above and mixed with 0.1 M imidazole in a ratio of 1:1. These mixtures were incubated for 2 h at 37°C, and the remaining FVIII:C was measured with a one-stage assay (Cephascreen reagents at the STAR Max3, both diagnostic Stago, Asnières sur Seine, France). Residual FVIII activity was defined as the percentage of remaining FVIII:C in the test plasma compared to the FVIII:C in the reference plasma. The original NBA formula was used to calculate inhibitor titer (9). A NBA titer of ≥ 0.6 NBU/mL was defined as positive (7). For samples containing emicizumab, the NBA was performed using bovine reagents, and the remaining FVIII:C was measured with a chromogenic assay (Siemens FVIII chromogenic reagents at the CS-2500 analyzer, Siemens Healthineers, Germany).

### Data analysis

SPSS version 26 was used to perform statistical analyses. Baseline characteristics were calculated using descriptive statistics. Continuous variables were reported as medians with an interquartile range (IQR). Categorical variables were presented as counts and percentages. The prevalence of NNA, very low-titer inhibitors, and inhibitors was calculated for the total population with all severities of hemophilia A. In a subgroup analysis, we compared antibody isotype and subclass and antibody titer levels between persons with NNA (divided into persons without inhibitor development and with a history of inhibitor development with and without previous immune tolerance induction treatment), very low-titer inhibitors, and inhibitors. In this subgroup analysis, we additionally analyzed hemophilia severity in terms of the number of cumulative exposure days to FVIII concentrates. The prevalence of NNA and neutralizing antibodies was compared between persons with null *F8* gene mutations comprising intron 22 inversion, intron 1 inversion, large deletions or duplications, nonsense mutations, and translocations, versus non-null *F8* gene mutations including small insertions or deletions, missense mutations, or splice-site mutations.

In a previous study, it was suggested that the presence of anti-FVIII IgA was associated with a history of hepatitis C virus (HCV) infection (12). Therefore, we compared the prevalence of FVIII-specific IgA isotype antibodies between persons with a history of HCV infection due to treatment with plasma products before 1992 and those without a HCV infection. The prevalence of IgA antibodies was compared between different clinical subgroups using Pearson’s Chi-square or Fischer’s exact test. *p*-values of ≤ 0.05 were considered statistically significant.

## Results

### Participant characteristics

**Figure 1:**
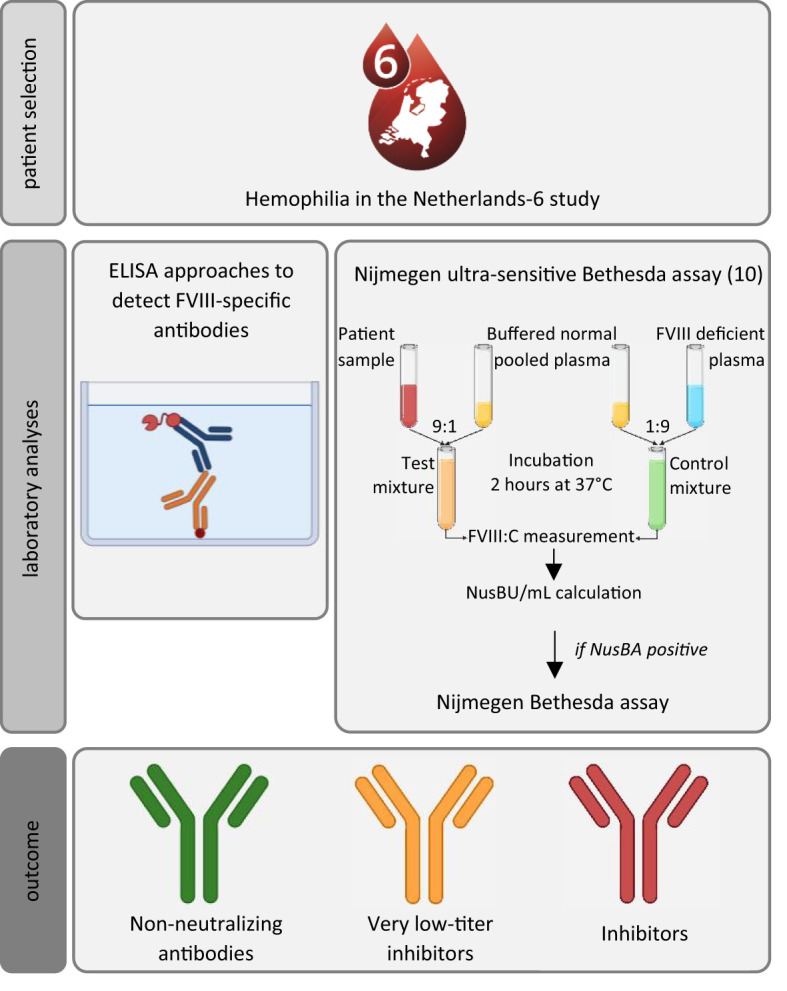
Schematic overview of the study methods to assess the prevalence and characteristics of non-neutralizing antibodies, very low-titer inhibitors, in our population. ELISA, enzyme-linked immunoassay; NusBA, Nijmegen ultra-sensitive Bethesda Assay. Participants were recruited from the *sixth Hemophilia in the Netherlands* cohort; all had an available plasma sample. Laboratory analysis consisted of ELISA approaches to detect FVIII-specific antibodies (direct-binding ELISA approaches were used to detect FVIII-binding antibodies and to assess the antibody titer levels, and FVIII specificity was confirmed by using a competition-based ELISA approach), and the Nijmegen ultra-sensitive Bethesda assay and Nijmegen Bethesda assay were used to detect very low-titer inhibitors or inhibitors. Icons were created using BioRender.

In total, 788 persons with congenital hemophilia A were recruited from the *sixth Hemophilia in the Netherlands* study (**Figure 1**). Of these, 336 (42.6%) persons had mild, 123 (15.6%) had moderate, and 329 (41.8%) had severe hemophilia A (**Table 1**). The median age was 45 years (IQR 24–60). In 41.1% of participants, FVIII prophylaxis was used, mostly (95.7%) a recombinant product. Overall, the majority (50.9%) had over 150 exposure days to FVIII concentrates.

**Table 1:** Clinical characteristics of patients in the study cohort.

Characteristics	Overall (*n* = 788)	Mild HA (*n* = 336)	Moderate HA (*n* = 123)	Severe HA (*n* = 329)
**Median age in years (IQR)**	45 (24–60)	51 (31–64)	39 (24–59)	36 (21–56)
Age categories (*n* (%))				
Children (0–17 years)	115 (14.6)	35 (10.4)	20 (16.3)	60 (18.2)
Adults (≥ 18 years)	673 (85.4)	301 (89.6)	103 (83.7)	269 (81.8)
Treatment regimen (*n* (%))				
On-demand	464 (58.9)	333 (99.1)	101 (82.1)	30 (9.1)
Prophylaxis	324 (41.1)	3 (0.9)	22 (17.9)	299 (90.9)
Cumulative exposure days to FVIII (*n* (%))				
< 50	310 (39.3)	265 (78.8)	37 (30.1)	8 (2.4)
50–150	77 (9.8)	43 (12.8)	27 (22.0)	7 (2.1)
> 150	401 (50.9)	28 (8.3)	59 (50.0)	314 (95.4)
Type of product used for prophylaxis (*n* (%))				
pdFVIII	2 (0.6)	1 (33.3)	0	1 (0.3)
Standard half-life rFVIII	258 (79.6)	2 (66.7)	20 (90.9)	236 (78.9)
Extended half-life rFVIII	52 (16.0)	0	2 (9.1)	50 (16.7)
aPCC	2 (0.6)	0	0	2 (0.7)
rFVIIa	1 (0.3)	0	0	1 (0.3)
Emicizumab	9 (2.8)	0	0	9 (3.0)
Inhibitor status (*n* (%))				
Never	680 (86.3)	314 (93.5)	108 (87.8)	258 (78.4)
Past	86 (10.9)	15 (4.5)	11 (8.9)	60 (18.2)
Current	16 (2.0)	5 (1.5)	2 (1.6)	9 (2.7)
Current or past^†^	6 (0.8)	2 (0.6)	2 (1.6)	2 (0.6)
Received immune tolerance induction to eradicate inhibitor (*n* (%))				
Yes	46 (42.6)	1 (4.5)	3 (20)	43 (60.6)
No	55 (50.9)	20 (90.9)	11 (73.3)	24 (33.8)
Unknown	7 (6.5)	1 (4.5)	1 (6.7)	4 (5.6)
HCV infection^a^ (*n* (%))				
Never	540 (68.5)	288 (85.7)	84 (68.3)	168 (51.1)
Past	236 (29.9)	46 (13.7)	38 (30.9)	152 (46.2)
Current	8 (1.0)	2 (0.6)	1 (0.8)	5 (1.5)
Current or past^b^	4 (0.5)	0	0	4 (1.2)

n, number; HA, hemophilia A; pdFVIII, plasma-derived factor VIII; rFVIII, recombinant factor VIII; aPCC, activated prothrombin complex concentrate; rFVIIa, recombinant activated factor VII; n.a., nonapplicable.

a All persons with a past or current HCV infection were treated with plasma products before 1992.

b For these participants, it could not be classified according to data retrieved from medical records or self-reported questionnaires.

### FVIII-binding and FVIII-specific antibodies

**Figure 2:**
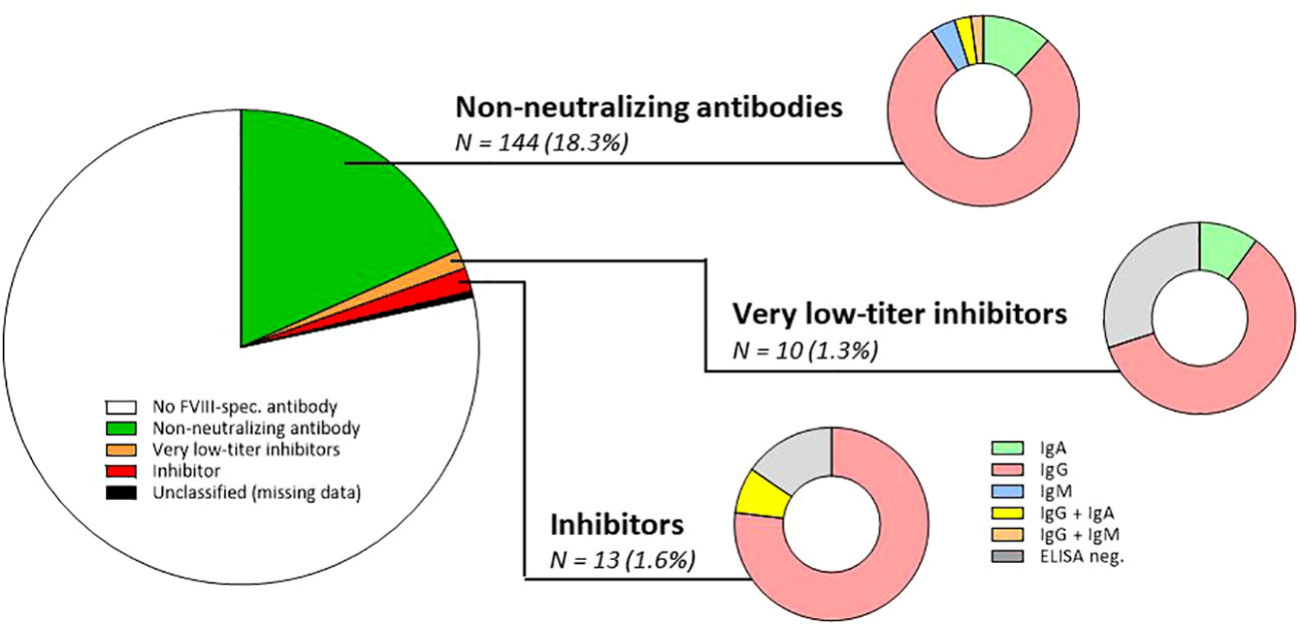
Prevalence of non-neutralizing FVIII-specific antibodies, very low-titer inhibitors, and inhibitors in the study population of 788 persons with haemophilia A. *N*, number; FVIII, factor VIII; ELISA, enzyme-linked immunoassay; IgA, immunoglobulin A; IgG, immunoglobulin G; IgM, immunoglobulin M. It should be mentioned that in the total cohort, 165 FVIII-specific antibodies were detected by ELISA. These 165 antibodies were classified as NNA (*n* = 144; both NusBA and NBA negative), very low-titer inhibitor (*n* = 7/10 ELISA positive, all NusBA positive), and inhibitor (*n* = 11/13 ELISA positive, all NusBA and NBA positive), and three persons with ELISA-positive results were unclassified due to missing NusBA and NBA data (*n* = 3). For non-neutralizing antibodies, the antibody isotype is composed of IgA (*n* = 17; 11.8%), IgG (*n* = 114; 79.2%), IgM (*n* = 6; 4.2%), IgG and IgA (*n* = 4; 2.8%), or IgG and IgM antibodies (*n* = 3; 2.1%). For very low-titer inhibitors, the antibody isotype included IgA (*n* = 1; 10.0%) and IgG (*n* = 6; 60.0%), and three (30.0%) persons were ELISA negative. For inhibitors, the antibody isotype included IgG (*n* = 10; 76.9%) and IgG and IgA antibodies (*n* = 1; 7.7%), and two (15.4%) persons were ELISA negative.

FVIII-binding antibodies were detected in 336 (42.6%) participants employing an ELISA approach, as described in the Methods section (**Figure 1**). As indicated in previous studies, it is crucial to perform competition studies to determine whether signals observed are specific for FVIII (15, 16). Competition assays confirmed FVIII-specificity in 165 individuals (20.9% of the total cohort) (**Figure 2**). The observed reduction in signal upon competition with excess FVIII was more effective in samples from persons with neutralizing antibodies as compared to those without neutralizing antibodies (**Supplementary Figure S2**).

### Prevalence and characteristics of NNA

Of persons with FVIII-specific antibodies, 144 (18.3% of the total cohort) had NNA (ELISA positive, NusBA, and NBA negative) (**Figure 2**; **Supplementary Table S4**). Antibody subclass analysis identified only IgG antibodies in 114 (79.2%), only IgM antibodies in six (4.2%), only IgA antibodies in 17 (11.8%), both IgG and IgM antibodies in three (2.1%), and both IgG and IgA in four (2.8%) participants (**Figure 2**). The most abundant antibody subclass was IgG1, which was present in 77 persons (53.5%) (**Supplementary Table S5**).

**Figure 3:**
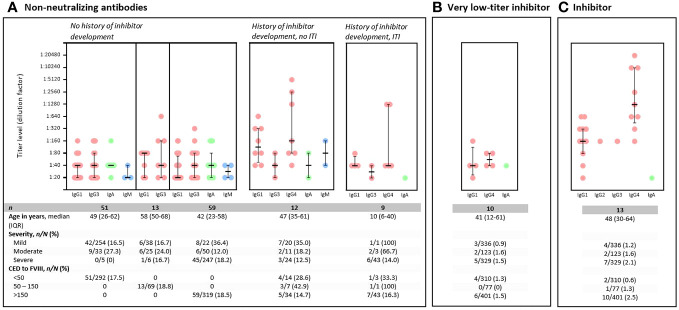
Characteristics of non-neutralizing FVIII-specific antibodies, very low-titer inhibitors, and inhibitors. y, year; *n*, number of participants; *N*, total number of participants; CED, cumulative exposure days; FVIII, factor VIII; ITI, immune tolerance induction. Titer levels are presented with a median and an interquartile range. Each dot represents one positive measurement. Some participants only have one antibody present, whereas others have multiple antibodies in their sample. For non-neutralizing antibodies **(A)**; antibody isotype and subclass and titer levels are presented according to different subgroups of participants. **(A)** Left panel: data of individuals without a history of inhibitor development, divided into different parts; data of persons with < 50 CED to FVIII (*n* = 51, left part); persons with 50–150 CED to FVIII (*n* = 13, middle part); and persons highly exposed to FVIII (CED > 150) (*n* = 59; right part). No IgG4 subclass antibodies were present in persons with NNA without a history of inhibitor development. For persons with non-neutralizing antibodies, the table presents data for 782 participants, as data on ITI therapy were missing for six participants (one mild (< 50 CED), one moderate (>150 CED), and four severe (> 150 CED) hemophilia A). Middle panel: NNA data of persons with a history of inhibitor development who did not receive ITI treatment. Right panel: NNA data for persons with a history of inhibitor development who did not receive ITI treatment. **(B)** Antibody titer levels obtained in ELISA approaches of seven (seven of 10) participants with very low-titer inhibitors, as the remaining three participants were ELISA negative (but NusBA positive). **(C)** Antibody titer levels obtained in ELISA approaches of 11 (11/13) participants with inhibitors, as the remaining two participants were ELISA negative (but NusBA and NBA positive).

NNA was present in a heterogeneous population. Therefore, in **Figure 3A**, we present NNA characteristics for different subgroups of participants. Antibody subclass and isotypes and antibody titers were shown for participants without a history of inhibitor development with less than 50, between 50 and 150, and over 150 cumulative exposure days to FVIII (**Figure 3A**, left panel), with a history of inhibitor development but without previous immune tolerance induction treatment (**Figure 3A**, middle panel), and those with inhibitor development with previous immune tolerance induction treatment (**Figure 3A**, right panel).

In persons without a history of inhibitor development, no clear differences were observed in antibody patterns between those who were minimally or highly exposed to FVIII concentrates (**Figure 3A**, left panel); only IgG1, IgG3, IgA, or IgM antibodies were detected, regardless of the number of exposure days to FVIII; no IgG4 subclass antibodies were detected (**Figure 3A**, left panel). In contrast, IgG4 subclass antibodies were detected in persons with a history of inhibitor development (with and without immune tolerance induction therapy) (**Figure 3A**, middle and right panels). In persons with a history of inhibitor development who received immune tolerance induction, no IgM subclass antibodies were detected (**Figure 3A**, right panel).

The overall median titer level for IgG1 subclass antibodies for persons with NNA without a history of inhibitor development was 1:40 (interquartile range (IQR) 1:20–1:80) (**Figure 3A**, left panel). Median titer levels for IgG1 subclass antibodies for persons with a history of inhibitor development without and with immune tolerance induction therapy were 1:120 (IQR 1:50–1:320) and 1:40 (IQR 1:40–1:70), respectively (**Figure 3A**, middle and right panels). Overall, the highest titer levels were detected for IgG4 subclass antibodies for persons with NNA with a history of inhibitor development (median 1:120; IQR 1:40–1:1,280) (**Figure 3A**, middle and right panels). The median titer levels of IgG4 subclass antibodies were 1:160 (IQR 1:80–1:2,560) for persons without immune tolerance induction therapy (**Figure 3A**, middle panel) and 1:40 (IQR 1:40–1:1,280) for those with immune tolerance induction therapy (**Figure 3A**, right panel).

There was no clear correlation between the *F8* genotype and the presence of NNA or neutralizing antibodies (*p* = 0.098) (**Supplementary Table S6**).

### Prevalence and characteristics of very low-titer FVIII inhibitors, as measured by NusBA

NusBA was performed in all participants, which was positive in 23 participants. In 10 of these 23 persons, NBA was negative, defining these antibodies as very low-titer inhibitors (10/788; 1.3% of the total cohort) (**Figure 2**; **Supplementary Table S7**). Of the 10 persons with very low-titer inhibitors, three (three of 10) had mild, two (two of 10) had moderate, and five (five of 10) had severe hemophilia A. Most (six of 10) had over 150 cumulative exposure days to FVIII. The median NusBA titer was 0.19 NusBU/mL (IQR 0.13–0.45).

Antibody subclass and isotype characteristics and antibody titer levels of the very low-titer inhibitors are presented in **Figure 3B** and **Supplementary Table S7**. Five persons had either IgG1 or IgG4 subclass antibodies, or both subclass antibodies were present. In one participant, IgA isotype antibodies were present.

Three participants, 145, 147, and 154, had positive NusBA results (0.13, 0.18, and 0.12 NusBU/mL, respectively), but no FVIII-binding IgG, IgM, or IgA antibodies were detected in ELISA (**Supplementary Tables S7, S10**). We would have expected a positive ELISA result in all NusBA-positive participants. For participant 145, ELISA results for the IgG4 subclass antibody were marginally positive; however, results were below the cut-off for FVIII specificity. For participants 154, ELISA results for IgG4 subclass antibodies were marginally positive in only 50% of experiments, which was defined as negative. However, for both participants 145 and 154, it is possible that very low-titers of IgG4 subclass antibodies caused the inhibition of FVIII in the NusBA. For the other participants 147, a possible explanation for the discrepancy between the ELISA and NusBA results is the use of a direct oral anticoagulant, which could cause false-positive NusBA results (22).

NusBA was not measured in three participants due to insufficient plasma material (**Supplementary Table S8**).

### Prevalence and characteristics of FVIII inhibitors, as measured by NBA

NBA was performed in all NusBA-positive participants, identifying FVIII inhibitors. NBA was positive in 13 out of 23 NusBA-positive persons (13/788; 1.6% of the total cohort) (**Figure 2**; **Supplementary Table S9**). Four (four of 13) persons with an inhibitor had mild, two (two of 13) had moderate, and seven (seven of 13) had severe hemophilia A. Most persons with an inhibitor (10/13) had over 150 cumulative exposure days to FVIII. The median NBA titer was 1.78 NBU/mL (IQR 0.94–6.38 NBU/mL).

Antibody subclass and isotype characteristics and antibody titer levels of the inhibitors are presented in **Figure 3C** and **Supplementary Table S9**. Of 11 persons, 10 (90.9%) had IgG1 and IgG4 subclass antibodies. The median titer level for IgG1 subclass antibodies was 1:160 (IQR 1:80–1:320), and the highest antibody titer levels were found for IgG4 subclass antibodies (median 1:1,280; IQR 1:480–1:10,240) (**Figure 3C**).

Two participants, 158 and 163, had positive NusBA and NBA results (> 0.8 NusBU/mL and 2.80 NBU/mL, and 0.33 NusBU/mL and 1.0 NBU/mL, respectively), but no FVIII-binding IgG, IgM, or IgA antibodies were detected in ELISA (**Supplementary Tables S9, S11**). We would have expected a positive ELISA result for all NBA-positive participants. We checked for the presence of antiphospholipid antibodies, which can interfere with NBA and cause false-positive results, but these were not detected. Another possible explanation for the discrepancy between ELISA and NBA results for participant 158 was that the ELISA results for the IgG1 subclass antibody were marginally positive, or just below the lower limit of detection, which was defined as negative. However, it is possible that very low titers of IgG1 subclass antibodies caused the inhibition of FVIII in the functional NBA assay. The discrepancy between ELISA and NBA results for participant 163 could not be explained.

### Prevalence of FVIII-specific IgA in clinical subgroups

FVIII-specific IgA antibodies were significantly more prevalent in persons with a history of HCV infection than in those without HCV infection (*p* = 0.009; **Supplementary Tables S12, S13**). FVIII-specific IgA antibody median titer levels were similar between persons with or without HCV infection (1:40, range 1:20–1:160). Age was not associated with the presence of FVIII-specific IgA antibodies (**Supplementary Table S12**). Prevalence of FVIII-specific IgA was not associated with other clinical characteristics (**Supplementary Table S12**). We additionally compared the prevalence of FVIII-specific IgG and IgM subclass and isotype antibodies between persons with and without a history of HCV infection, which was not statistically significantly different between the two groups (**Supplementary Table S13**).

## Discussion

In a large nationwide population of 788 persons with hemophilia A of all severities, we investigated the prevalence and characteristics of FVIII-specific NNA, very low-titer inhibitors (assessed with the recently developed NusBA), and inhibitors (assessed with the NBA). We identified the full spectrum of anti-FVIII antibodies, with a prevalence of 18.3% NNA, 1.3% very low-titer inhibitors that were only detected by the NusBA, and 1.6% FVIII inhibitors detected by the NBA.

It has been hypothesized that the anti-FVIII immune response is a continuum between non-neutralizing and neutralizing antibodies. Several studies found NNA titer and IgG subclass signatures to be associated with subsequent inhibitor development (16, 18). In line with our results, previous research showed that antibodies of the IgG1 subclass were most prevalent in persons with NNA (12, 15). In fact, the HIPS study found IgG1 antibodies to be the only IgG subclass present in both persons with NNA and persons with inhibitors and therefore evaluated the characteristics of these FVIII-specific IgG1 antibodies in both subgroups. In their study, IgG1 antibody titers were significantly higher in persons with persistent inhibitors (median 1:1,280; IQR 1:320–1:61,440), compared to persons with NNA (median 1:40; IQR 1:30–1:80) (18). In accordance, we also found a higher median IgG1 titer in persons with current inhibitors (1:160; IQR 1:80–1:320) compared to persons with NNA without a history of inhibitor development (1:40; IQR 1:20–1:80). Furthermore, the HIPS study demonstrated that the appearance of IgG3 subclass antibodies, with the subsequent development of IgG4 antibodies, was associated with the development of persistent inhibitors (18). However, as also shown by Whelan et al., we rather found a predominance of IgG1 and IgG4 (16). After inhibitor eradication, low levels of FVIII-specific non-neutralizing antibodies may potentially persist that are unable to neutralize FVIII. Indeed, we found lower titer levels of IgG1 and IgG4 subclass antibodies were detected in persons with NNA with a history of inhibitor development with immune tolerance induction therapy (1:40; IQR 1:40–1:70 and 1:40; IQR 1:40–1:1,280, respectively), as compared to those with a current inhibitor (1:320; IQR 1:80–1:320 and 1:1,280; IQR 1:480–1:1,024, respectively) (**Figures 3A** (right panel), **3C**). It has been speculated that persistently low levels of FVIII-specific NNA might even be important in the maintenance of immune tolerance through their interaction with the inhibitory FcγRIIB receptor on plasma cells (23).

We theoretically expected to find FVIII-specific antibodies in all persons with a very low-titer inhibitor. Remarkably, we did not detect those antibodies in three persons with a very low-titer inhibitor and two persons with an inhibitor. We cannot fully exclude that residual (exogenous) FVIII has formed FVIII immune complexes with present antibodies, which may have impeded the detection of these antibodies by ELISA. In the ELISA setup used, we directly immobilized FVIII on microtiter wells. Potentially, this might shield the binding of anti-FVIII antibodies to functional epitopes that are targeted by a subset of neutralizing antibodies. We have employed a full-length FVIII for the detection of anti-FVIII antibodies by ELISA. We cannot fully exclude that the presence of the B domain may interfere with the binding of anti-FVIII antibodies to immobilized FVIII in a subset of patient samples. For NusBA and NBA assays, preanalytical variables as well as nonantibody components such as anticoagulants present in the plasma may potentially interfere with assay results. Furthermore, as NusBA incorporates 90% patient plasma and 10% normal control plasma and readout takes place in the lower range of the FVIII chromogenic assay, it may hypothetically be more sensitive to interference compared to NBA, in which the proportion of patient plasma/normal control plasma is 50%/50% and readout is in the median assay range (10). Preanalytical deviations have been excluded as much as possible by using a uniform blood collection protocol. We have diminished the risk of interference as much as possible by reporting the use of anticoagulant medication in NusBA-/NBA-positive and ELISA-negative samples. Furthermore, the presence of lupus anticoagulants could potentially lead to false detection of an FVIII inhibitor in NBA one-stage coagulation assays, causing a positive result in the NBA (24). However, no antiphospholipid antibodies were detected in the two NBA-positive and ELISA-negative samples.

Previous research by Paul and colleagues hypothesized that HCV infection might foster a FVIII-specific IgA antibody response (12). Our results also showed a higher prevalence of FVIII-specific IgA antibodies in persons with a history of HCV infection than in those without (5.2% versus 1.9%; *p* = 0.009). We assessed whether the IgA prevalence was different according to age, as patients with a history of HCV infection may be older than those without a history of HCV infection. However, it appeared that age was not associated with the presence of FVIII-specific IgA antibodies. Ahmadi and colleagues proposed that it may be possible that antibodies in persons with advanced liver disease caused by hepatitis B or C virus infection can be cross-reactive against both bacterial antigens and human proteins (25). We speculate that HCV infection may promote the development of autoreactive IgA antibodies that cross-react with FVIII. Nevertheless, the clinical relevance of anti-FVIII IgA antibodies is currently unclear since anti-FVIII IgA are not clearly linked to FVIII inhibitors (18).

This is the first study investigating the full spectrum of FVIII-specific antibodies, including both non-neutralizing antibodies as well as very low-titer inhibitors, in a large population of persons with hemophilia A of all severities. A strength of this study is that we incorporated a very sensitive and specific ELISA approach to detect FVIII-binding antibodies, and moreover, we evaluated FVIII specificity in all samples with positive FVIII-binding antibodies. In addition, we were able to detect very low-titer inhibitors by the novel sensitive NusBA (10). We were unable to evaluate whether NNA evolved into very low-titer inhibitors and inhibitors due to the cross-sectional study design of this study. In addition, we were unable to assess the affinity of the antibodies. The previously mentioned difference in signal reduction upon FVIII competition in the FVIII-specific ELISA is hypothesized to be due to differences in affinity of the antibodies for FVIII, as it is postulated neutralizing antibodies may have a higher affinity for FVIII, as compared to non-neutralizing antibodies. Furthermore, Advate^®^ has previously been shown to be highly similar with respect to glycosylation when compared to wild-type FVIII (26). Nevertheless, we indeed cannot fully exclude that antibodies may exist that are directed toward post-translational modifications, which are not present on Advate^®^ but that are present on other FVIII products. In addition, in persons with severe hemophilia, cross-reactive material is rarely present, and if it is present, the concentration is generally very low. In persons with nonsevere hemophilia, cross-reactive material is generally present, but the vast majority of individuals will be tolerant of this endogenous FVIII protein. Unfortunately, it was not feasible to address the impact of cross-reactive material on the anti-FVIII antibody titers as measured by ELISA, since it is technically not possible to separate circulating FVIII-anti-FVIII antibodies in small volumes of patient samples. To circumvent this issue, we have coated plates with large amounts of recombinant FVIII (1 μg/ml), which we expect will compete efficiently with the low levels of endogenous FVIII that are present in cross-reactive material-positive patient samples. Nevertheless, there is a theoretical possibility that titers of anti-FVIII antibodies may be lower or even below the limit of detection in samples containing cross-reactive material. Furthermore, we cannot fully exclude that healthy individuals with FVIII-binding antibodies were present in our plasma pool of 40 healthy donors used as a negative control in ELISA. However, the very low OD values of our negative controls indicate that no or very limited antibodies were present in the pool. Also, our antibody prevalence is comparable to previously published data; therefore, we assume that we have not missed the detection of antibodies in our cohort. At last, it should be noted that the prevalence of current inhibitors in our population is relatively low. This could be attributed to the relatively older study population, which includes a significant proportion of persons highly exposed to FVIII concentrates. Furthermore, in the Netherlands, almost all participants with inhibitors are treated with immune tolerance induction therapy. Therefore, the majority of our population represents individuals tolerant to FVIII.

Based on our current findings, it will be crucial to evaluate in future studies which NNAs and very low-titer inhibitors will disappear, retain, or even evolve into clinically relevant inhibitors over time. In view of the high frequency of NNA in our study population, we anticipate that a considerable number of persons with hemophilia will respond to treatment by developing low levels of anti-FVIII antibodies detectable by ELISA. Apparently, these low levels of anti-FVIII antibodies do not further develop into clinically relevant inhibitors in the majority of patients, who are most likely already established as tolerant against FVIII. Whether an antibody will develop into a clinically relevant inhibitor, may be determined by the type of interaction that is generated by immune cells. The immune response generating non-neutralizing IgG1 FVIII-specific antibodies may be stopped in an early, nonadvanced activation phase of FVIII-specific B-cell responses, as compared to the immune response generating neutralizing antibodies. B-cell activation in response to FVIII could be restrained when second signals, such as signals provided by cognate interactions between FVIII-specific helper T cells, are limiting (18, 27). We speculate that insufficient help by CD4^+^ T cells and the absence of costimulatory signals provoked by inflammation and/or bleeding prevent further progression of the immune response in these patients. In line with this, only a very small proportion of NNA is of subclass IgG4, only in persons with an inhibitor history. Generation of anti-FVIII antibodies of subclass IgG4 requires CD4^+^ T cell-dependent class switching (28). Conversely, the prevalence of anti-FVIII IgG4 is much higher in persons with very low-titer inhibitors and inhibitors, as measured by NusBA and NBA (**Figure 3**). Based on these results, we propose that the sensitive ELISA-based method for the detection of anti-FVIII antibodies of subclass IgG4 described in this study can potentially be used as a predictive biomarker for the development of clinically relevant inhibitors. The inclusion of the very sensitive NusBA may further facilitate the early detection of patients at risk of developing clinically relevant inhibitors. It should be noted that based on our results, we cannot fully exclude that the underlying mechanism generating either NNA or neutralizing antibodies may be different. The molecular regulation of early B-cell effector responses, such as the endogenous brakes in antibody class-switch recombination and B-cell differentiation into antibody-secreting cells may play an important role (18). Future longitudinal follow-up of anti-FVIII antibodies at the clonal level are needed to establish that NNA indeed develops into high-affinity inhibitory anti-FVIII antibodies in a CD4^+^ T cell-dependent manner, as is suggested by the currently available literature on FVIII inhibitors (27).

Currently, nonfactor therapies such as emicizumab are increasingly used as prophylaxis. FVIII is still needed for the treatment of breakthrough bleeds in those patients receiving emicizumab. Exposure to relatively large doses of FVIII in the context of bleeding may potentially provoke inhibitor development. Regular monitoring of FVIII-specific antibodies as well as the very low-titer inhibitors by using the NusBA may help to identify patients at risk for developing inhibitors and tailor treatment based on the antibody spectrum present in persons with hemophilia A.

In conclusion, in our cross-sectional study consisting of 788 persons, we identified non-neutralizing FVIII-specific antibodies in a relatively large proportion of a nationwide population of persons with all severities of hemophilia A. Interestingly, IgG4 subclass antibodies were only observed in persons with a reported history of FVIII inhibitor and in those with a current (very-low titer) inhibitor. In our population, consisting of relatively older persons highly exposed to FVIII concentrates, only a small proportion (very low-titer) of inhibitors were detected, reflecting a tolerized population.

For future research, especially in persons treated with emicizumab or other nonfactor therapies who are still at risk of inhibitor development, we propose to longitudinally study the development and characteristics of NNA, very low-titer inhibitors, and inhibitors. We would suggest following up with these persons after FVIII treatment employing both the functional and nonfunctional tests outlined in this study to monitor for the occurrence of FVIII-specific antibodies, including their characteristics (NNA, very low-titer inhibitors, and inhibitors, as well as isotypes IgA, IgM, and IgG subclasses), to determine whether the development of NNA plays a role in establishing or maintaining tolerance to FVIII.

## Data availability statement

The raw data supporting the conclusions of this article will be made available by the authors, without undue reservation.

## Ethics statement

The studies involving humans were approved by Medical Ethical Committee of Leiden University Medical Center (registration number NL59144.058.17). The studies were conducted in accordance with the local legislation and institutional requirements. Written informed consent for participation in this study was provided by the participants’ legal guardians/next of kin.

## Author contributions

IO: Data curation, Formal analysis, Investigation, Methodology, Project administration, Writing – original draft, Writing – review & editing. MV: Data curation, Formal analysis, Investigation, Methodology, Project administration, Writing – original draft, Writing – review & editing. MM: Resources, Writing – review & editing. PA: Methodology, Writing – review & editing. EB: Resources, Writing – review & editing. NB: Writing – review & editing. JB: Writing – review & editing. MC: Resources, Writing – review & editing. MD: Writing – review & editing. JE: Resources, Writing – review & editing. KF: Funding acquisition, Supervision, Writing – review & editing. SH: Resources, Writing – review & editing. WH: Writing – review & editing. HLH: Resources, Writing – review & editing. JJ: Writing – review & editing. PK: Methodology, Writing – review & editing. FL: Resources, Writing – review & editing. DM: Formal analysis, Writing – review & editing. HP: Methodology, Writing – review & editing. SR: Formal analysis, Methodology, Writing – review & editing. FR: Writing – review & editing. CS: Writing – review & editing. LV: Resources, Writing – review & editing. JV: Investigation, Methodology, Supervision, Writing – original draft, Writing – review & editing. SS: Investigation, Methodology, Supervision, Writing – original draft, Writing – review & editing. SG: Investigation, Methodology, Supervision, Writing – original draft, Writing – review & editing.

## References

[1] Srivastava A Santagostino E Dougall A Kitchen S Sutherland M Pipe SW. WFH guidelines for the management of hemophilia, 3rd edition. Haemophilia (2020) 26 Suppl 6:1–158. doi: 10.1111/hae.14046 32744769

[2] Peyvandi F Garagiola I Young G. The past and future of haemophilia: diagnosis, treatments, and its complications. Lancet (2016) 388(10040):187–97. doi: 10.1016/S0140-6736(15)01123-X 26897598

[3] Walsh CE Jiménez-Yuste V Auerswald G Grancha S. The burden of inhibitors in haemophilia patients. Thromb Haemost (2016) 116 Suppl 1:S10–7. doi: 10.1160/TH16-01-0049 27528280

[4] Gouw SC van den Berg HM Fischer K Auerswald G Carcao M Chalmers E. Intensity of factor VIII treatment and inhibitor development in children with severe hemophilia A: the RODIN study. Blood (2013) 121(20):4046–55. doi: 10.1182/blood-2012-09-457036 23553768

[5] Wight J Paisley S. The epidemiology of inhibitors in haemophilia A: a systematic review. Haemophilia (2003) 9(4):418–35. doi: 10.1046/j.1365-2516.2003.00780.x 12828678

[6] Eckhardt CL Loomans JI van Velzen AS Peters M Mauser-Bunschoten E Schwaab R. Inhibitor development and mortality in non-severe hemophilia A. J Thromb Haemost (2015) 13(7):1217–25. doi: 10.1111/jth.12990 25912309

[7] Blanchette VS Key NS Ljung LR Manco-Johnson MJ van den Berg HM Srivastava A. Definitions in hemophilia: communication from the SSC of the ISTH. J Thromb Haemost (2014) 12(11):1935–9. doi: 10.1111/jth.12672 25059285

[8] Giles AR Verbruggen B Rivard GE Teitel J Walker I. A detailed comparison of the performance of the standard versus the Nijmegen modification of the Bethesda assay in detecting factor VIII:C inhibitors in the haemophilia A population of Canada. Association of Hemophilia Centre Directors of Canada. Factor VIII/IX Subcommittee of Scientific and Standardization Committee of International Society on Thrombosis and Haemostasis. Thromb Haemost (1998) 79(4):872–5. 9569207

[9] Verbruggen B Novakova I Wessels H Boezeman J van den Berg M Mauser-Bunschoten E. The Nijmegen modification of the Bethesda assay for factor VIII:C inhibitors: improved specificity and reliability. Thromb Haemost (1995) 73(2):247–51. doi: 10.1055/s-0038-1653759 7792738

[10] Valke LLFG Verhagen MJA Mulders BTPM Polenewen R Blijlevens NMA Jansen JH. The Nijmegen ultra-sensitive Bethesda Assay detects very low-titer factor VIII inhibitors in patients with congenital and acquired hemophilia A. Thromb Res (2023) 231:112–20. doi: 10.1016/j.thromres.2023.10.007 37844518

[11] Hofbauer CJ Kepa S Schemper M Quehenberger P Reitter-Pfoertner S Mannhalter C. FVIII-binding IgG modulates FVIII half-life in patients with severe and moderate hemophilia A without inhibitors. Blood (2016) 128(2):293–6. doi: 10.1182/blood-2015-10-675512 27216215

[12] Schweiger H Rejtő J Hofbauer CJ Berg V Allacher P Zwiauer K. Nonneutralizing FVIII-specific antibody signatures in patients with hemophilia A and in healthy donors. Blood Adv (2022) 6(3):946–58. doi: 10.1182/bloodadvances.2021005745 PMC8945293 34847225

[13] Battle J Gómez E Rendal E Torea J Lourés E Couselo M. Antibodies to factor VIII in plasma of patients with hemophilia A and normal subjects. Ann Hematol (1996) 72(5):321–6. doi: 10.1007/s002770050179 8645745

[14] Pratt KP Gunasekera D Vir P Tan S Pierce GF Olsen CH. Anti-FVIII antibodies in Black and White hemophilia A subjects: do F8 haplotypes play a role? Blood Adv (2022) 7(17):4983–98. doi: 10.1182/bloodadvances.2021004909 PMC10471934 36459498

[15] Abdi A Bordbar MR Hassan S Rosendaal FR van der Bom JG Voorberg J. Prevalence and incidence of non-neutralizing antibodies in congenital hemophilia A— A systematic review and meta-analysis. Front Immunol (2020) 11(563). doi: 10.3389/fimmu.2020.00563 PMC7221178 32457734

[16] Whelan SF Hofbauer CJ Horling FM Allacher P Wolfsegger MJ Oldenburg J. Distinct characteristics of antibody responses against factor VIII in healthy individuals and in different cohorts of hemophilia A patients. Blood (2013) 121(6):1039–48. doi: 10.1182/blood-2012-07-444877 23243272

[17] Cannavò A Valsecchi C Garagiola I Palla R Mannucci PM Rosendaal FR. Nonneutralizing antibodies against factor VIII and risk of inhibitor development in severe hemophilia A. Blood (2017) 129(10):1245–50. doi: 10.1182/blood-2016-06-720086 28034891

[18] Reipert BM Gangadharan B Hofbauer CJ Berg V Schweiger H Bowen J. The prospective Hemophilia Inhibitor PUP Study reveals distinct antibody signatures prior to FVIII inhibitor development. Blood Adv (2020) 4(22):5785–96. doi: 10.1182/bloodadvances.2020002731 PMC7686884 33232473

[19] Hassan S van Balen EC Smit C Mauser-Bunschoten EP van Vulpen LFD Eikenboom J. Health and treatment outcomes of patients with hemophilia in the Netherlands, 1972-2019. J Thromb Haemost (2021) 19(10):2394–406. doi: 10.1111/jth.15424 PMC8518083 34117710

[20] van Den Brink EN Turenhout EA Davies J Bovenschen N Fijnvandraat K Ouwehand WH. Human antibodies with specificity for the C2 domain of factor VIII are derived from VH1 germline genes. Blood (2000) 95(2):558–63. doi: 10.1182/blood.V95.2.558 10627462

[21] van Helden PMW Berg van den HM Gouw SC Kraijen PHP Zuurveld MG Mauser-Bunschoten EP. IgG subclasses of anti-FVIII antibodies during immune tolerance induction in patients with hemophilia A. Br J Haematol (2008) 142:644–52. doi: 10.1111/j.1365-2141.2008.07232.x 18510679

[22] Moser KA Smock KJ. Direct oral anticoagulant (DOAC) interference in hemostasis assays. Hematol Am Soc Hematol Educ Program (2021) 1:129–33. doi: 10.1182/hematology.2021000241 PMC8791165 34889400

[23] Paul H Berg V Gangadharan B Bowen J LeBeau P Blatny J. Prospective hemophilia inhibitor PUP study reveals distinct antibody signatures during FVIII inhibitor eradication. Blood Adv (2022) 7(9):1831–48. doi: 10.1182/bloodadvances.2022007267 PMC10165197 36074992

[24] Adcock DM Favaloro EJ. Pearls and pitfalls in factor inhibitor assays. Int J Lab Hematol (2015) 37 Suppl 1:52–60. doi: 10.1111/ijlh.12352 25976961

[25] Ahmadi AR Song G Gao T Ma J Han X Hu M. Discovery and characterization of cross-reactive intrahepatic antibodies in severe alcoholic hepatitis. bioRxiv (2023). doi: 10.1101/2023.02.23.529702 PMC10699809 38055614

[26] Lai JD Swystun LL Cartier D Nesbitt K Zhang C Hough C. N-linked glycosylation modulates the immunogenicity of recombinant human factor VIII in hemophilia A mice. Haematologica (2018) 103(1):1925–36. doi: 10.3324/haematol.2018.188219 PMC6278987 30002126

[27] Lacroix-Desmazes S Voorberg J Lillicrap D Scott DW Pratt KP. Tolerating factor VIII: recent progress. Front Immunol (2020) 10:2991. doi: 10.3389/fimmu.2019.02991 31998296 PMC6965068

[28] Jing W Chen J Cai Y Chen Y Schroeder J Johnson BD. Induction of activated T follicular helper cells is critical for anti-FVIII inhibitor development in hemophilia A mice. Blood Adv (2019) 3(20):3099–110. doi: 10.1182/bloodadvances.2019000650 PMC6849959 31648333

