# Supplementary material for: The spectrum of neutralizing and non-neutralizing anti-FVIII antibodies in a nationwide cohort of 788 persons with hemophilia A
Source: Front Immunol. 2024 Feb 22;15:1355813. doi: 10.3389/fimmu.2024.1355813 (PMC10918462; doi:10.3389/fimmu.2024.1355813)
Supplement: Supplementary file 1 [file DataSheet_1.docx]

**Supplementary material of “The spectrum of neutralizing and non-neutralizing anti-FVIII antibodies in a nationwide cohort of 788 persons with hemophilia A”**

Ilja Oomen^†^ ^1,2^, Marieke Verhagen^†^ ^3,4,5^, Mariarosaria Miranda^2^, Peter Allacher^6^, Erik A. M. Beckers^7^, Nicole M.A. Blijlevens^3,4^, Johanna G. van der Bom^8^, Michiel Coppens^9^, Mariëtte Driessens^10^, Jeroen C.J. Eikenboom^11^, Karin Fijnvandraat^1,2^, Shermarke Hassan^8,12^, Waander. L. van Heerde^3,4,13^, H. Louise Hooimeijer^14^, Joop H. Jansen^5^, Paul Kaijen^2^, Frank W. G. Leebeek^15^, Daniëlle Meijer^5^, Helmut Paul^6^, Sanna R. Rijpma^5^, Frits R. Rosendaal^11^, Cees Smit^9,11^, Lize F. D. van Vulpen^16^, Jan Voorberg^2,8^, Saskia E.M. Schols^† 3,4^, Samantha C. Gouw^† 1,8^

^†^ These authors contributed equally to this work and share first or last authorship.

**Affiliations**

1. Department of Pediatric Hematology, Amsterdam UMC location University of Amsterdam, Amsterdam, the Netherlands
2. Department of Molecular Hematology, Sanquin Research, Amsterdam, the Netherlands
3. Department of Hematology, Radboud university medical center, Nijmegen, the Netherlands
4. Hemophilia Treatment Center Nijmegen-Eindhoven-Maastricht, Nijmegen, the Netherlands
5. Laboratory of Hematology, Department of Laboratory Medicine, Radboud university medical center, Nijmegen, the Netherlands
6. Institute Krems Bioanalytics, IMC University of Applied Sciences Krems, Krems, Austria
7. Division of Hematology, Department of Internal Medicine, Maastricht University Medical Center, Maastricht University, Maastricht, the Netherlands
8. Department of Clinical Epidemiology, Leiden University Medical Center, Leiden, the Netherlands
9. Department of Vascular Medicine, Amsterdam Cardiovascular Sciences, Amsterdam University medical Centers, location University of Amsterdam, Amsterdam, the Netherlands
10. Dutch Society of Hemophilia Patients, the Netherlands
11. Division of Thrombosis and Hemostasis, Department of Internal Medicine, Leiden University Medical Center, Leiden University, Leiden, the Netherlands
12. Infectious Diseases Data Observatory, Center for Tropical Medicine and Global Health, University of Oxford, Oxford, United Kingdom
13. Enzyre BV, Novio Tech Campus, Nijmegen, the Netherlands
14. Division of Hematology/Oncology, Department of Pediatrics, University Medical Center Groningen, University of Groningen, Groningen, the Netherlands
15. Department of Hematology, Erasmus University Medical Center, Rotterdam, the Netherlands
16. Center for Benign Hematology, Thrombosis and Haemostasis, Van Creveldkliniek, University Medical Center Utrecht, University Utrecht, Utrecht, the Netherlands

**Methods**

**M1. *Sixth Hemophilia in the Netherlands* blood collection protocol**

Protocol version 1.0.

Date 13 November 2017.

| **Table 1. Body materials to be collected by age group** | | | |
| --- | --- | --- | --- |
| **Age** | **Total amount of blood/urine** | **Tubes to be withdrawn** | **Amount of body material for storage** |
| 0-3 years | 7.2 mL blood | 1 x 4.5 mL citrate tube | 2.25 mL citrate plasma, DNA* |
|  |  | 1 x 2.7 mL citrate tube | 1.35 mL citrate plasma, DNA* |
|  | 10 mL urine | 1 x 10 mL urine tube | 5.4 mL urine after centrifuge |
| 4-9 years | 9 mL blood | 1 x 4.5 mL citrate tube | 2.25 mL citrate plasma, DNA* |
|  |  | 1 x 4.5 mL citrate tube | 2.25 mL citrate plasma, DNA* |
|  | 10 mL urine | 1 x 10 mL urine tube | 5.4 mL urine after centrifuge |
| 10-11 years | 17.5 mL blood | 1 x 4.5 mL citrate tube | 2.25 mL citrate plasma, DNA* |
|  |  | 2 x 4.5 mL citrate tube | 4.5 mL citrate plasma, DNA* |
|  |  | 1 x 4 mL serum tube | 2 mL serum |
|  | 10 mL urine | 1 x 10 mL urine tube | 5.4 mL urine after centrifuge |
| ≥12 years | 55 mL blood | 3 x 4.5 mL citrate tube | 6.75 mL citrate plasma, DNA* |
|  |  | 7 x 4.5 mL citrate tube | 15.5 mL citrate plasma, DNA* |
|  |  | 2 x 5 mL serum tube | 5 mL serum |
|  | 10 mL urine | 1 x 10 mL urine tube | 5.4 mL urine after centrifuge |
| *** DNA is isolated from the residue of citrate tubes after centrifuge the citrate plasma. | | | |

| **Table 2. Processing and storage of body material** | |  |  |
| --- | --- | --- | --- |
| **Material** | **Processing** | | **Storage** |
| Citrate plasma | 2 x 15 minutes centrifuge 3000g at room temperature | | -80° Celsius, aliquots of 0.5 mL |
| Serum | According to biobank protocol* | | -80° Celsius, aliquots of 0.5 mL |
| Urine | 10 minutes centrifuge at 1500g at 4° Celsius | | -80° Celsius, aliquots of 0.5 mL |
| DNA* | According to biobank protocol* | | Stock solution is stored at +4° Celsius or ≤20° Celsius |
|  | | | |

**Tables**

| **Table S1.** Horseradish peroxidase-conjugated anti-human antibodies used in ELISA. | | | |
| --- | --- | --- | --- |
| **Immunoglobulin isotype and subclass** | **Clone** | **Manufacturer** | **Dilution** |
| IgG1 | HP6188 | Sanquin | 1:10.000 |
| IgG2 | HP6002 | Southern Biotech | 1:10.000 |
| IgG3 | HP6050 | Cell Sciences | 1:50.000 |
| IgG4 | HP6025 | Southern Biotech | 1:25.000 |
| IgM | n.a. | Southern Biotech | 1:100.000 |
| IgA | n.a. | Bethyl | 1:100.000 |
| *Abbreviations:*  ELISA = enzyme-linked immunosorbent assay, IgG = immunoglobulin G, IgM = immunoglobulin M, IgA = immunoglobulin A, n.a. = not applicable. | | | |

| **Table S2.** Confirmation specificity and cross-reactivity of detection antibodies to their appropriate human immunoglobulin isotype and subclass. | | | | | | | | | | | | |
| --- | --- | --- | --- | --- | --- | --- | --- | --- | --- | --- | --- | --- |
| (1) | **Anti-IgG1** | | **Anti-IgG2** | | **Anti-IgG3** | | **Anti-IgG4** | | **Anti-IgA** | | **Anti-IgM** | |
| **IgG1** | 0,236 | 0,246 | 0,029 | 0,032 | 0,014 | 0,011 | 0,015 | 0,011 | 0,011 | 0,068 | 0,006 | 0,008 |
| **IgG2** | 0,008 | 0,008 | 0,365 | 0,386 | 0,006 | 0,009 | 0,009 | 0,013 | 0,015 | 0,026 | 0,009 | 0,008 |
| **IgG3** | 0,013 | 0,009 | 0,037 | 0,034 | 1,998 | 1,995 | 0,028 | 0,034 | 0,011 | 0,014 | 0,008 | 0,012 |
| **IgG4** | 0,010 | 0,016 | 0,033 | 0,034 | 0,026 | 0,013 | 2,027 | 2,035 | 0,008 | 0,011 | 0,008 | 0,008 |
| **IgA1** | 0,011 | 0,007 | 0,015 | 0,025 | 0,008 | 0,008 | 0,008 | 0,008 | 1,274 | 1,254 | 0,008 | 0,006 |
| **IgA2** | 0,009 | 0,008 | 0,015 | 0,017 | 0,010 | 0,007 | 0,041 | 0,008 | 0,966 | 0,920 | 0,008 | 0,009 |
| **IgM** | 0,009 | 0,010 | 0,016 | 0,020 | 0,019 | 0,011 | 0,012 | 0,009 | 0,073 | 0,047 | 1,745 | 1,750 |
| **Buffer** | 0,008 | 0,007 | 0,023 | 0,019 | 0,007 | 0,022 | 0,014 | 0,009 | 0,015 | 0,009 | 0,009 | 0,008 |

| (2) | **Anti-IgG1** | | **Anti-IgG2** | | **Anti-IgG3** | | **Anti-IgG4** | | **Anti-IgA** | | **Anti-IgM** | |
| --- | --- | --- | --- | --- | --- | --- | --- | --- | --- | --- | --- | --- |
| **IgG1** | 0,243 | 0,238 | 0,029 | 0,028 | 0,008 | 0,009 | 0,012 | 0,010 | 0,020 | 0,030 | 0,008 | 0,012 |
| **IgG2** | 0,008 | 0,008 | 0,343 | 0,356 | 0,011 | 0,009 | 0,010 | 0,009 | 0,015 | 0,050 | 0,007 | 0,013 |
| **IgG3** | 0,009 | 0,009 | 0,034 | 0,032 | 2,033 | 2,013 | 0,036 | 0,035 | 0,014 | 0,015 | 0,009 | 0,012 |
| **IgG4** | 0,007 | 0,008 | 0,036 | 0,035 | 0,021 | 0,012 | 2,029 | 2,031 | 0,017 | 0,013 | 0,011 | 0,009 |
| **IgA1** | 0,009 | 0,008 | 0,018 | 0,025 | 0,008 | 0,007 | 0,009 | 0,009 | 1,326 | 1,308 | 0,025 | 0,009 |
| **IgA2** | 0,009 | 0,008 | 0,031 | 0,021 | 0,012 | 0,008 | 0,009 | 0,008 | 0,990 | 0,969 | 0,009 | 0,012 |
| **IgM** | 0,008 | 0,008 | 0,019 | 0,015 | 0,009 | 0,013 | 0,013 | 0,010 | 0,053 | 0,035 | 1,807 | 1,785 |
| **Buffer** | 0,007 | 0,008 | 0,024 | 0,020 | 0,008 | 0,011 | 0,017 | 0,012 | 0,020 | 0,015 | 0,007 | 0,009 |

|  | **Anti-IgG1** | | **Anti-IgG2** | | **Anti-IgG3** | | **Anti-IgG4** | | **Anti-IgA** | | **Anti-IgM** | |
| --- | --- | --- | --- | --- | --- | --- | --- | --- | --- | --- | --- | --- |
| **IgG1** |  |  |  |  |  |  |  |  |  |  |  |  |
| **IgG2** |  |  |  |  |  |  |  |  |  |  |  |  |
| **IgG3** |  |  |  |  |  |  |  | 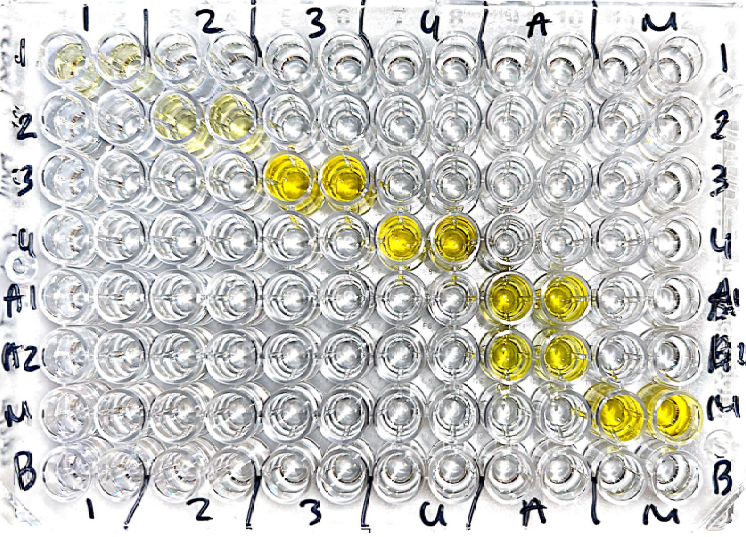 |  |  |  |  |
| **IgG4** |  |  |  |  |  |  |  |  |  |  |  |  |
| **IgA1** |  |  |  |  |  |  |  |  |  |  |  |  |
| **IgA2** |  |  |  |  |  |  |  |  |  |  |  |  |
| **IgM** |  |  |  |  |  |  |  |  |  |  |  |  |
| **Buffer** |  |  |  |  |  |  |  |  |  |  |  |  |

| **Table S3.** Overview of third repetitions performed for the titration ELISAs, because of a larger variation than one dilution step between two duplicate assays. | | | | |
| --- | --- | --- | --- | --- |
| **ID** | **First assay**  **FVIII-specific antibody titer**  **(dilution step)** | **Second assay**  **FVIII-specific antibody titer (dilution step)** | **Third repetition**  **FVIII-specific antibody titer (dilution step)** | **Reported titer** |
| **IgG1** | | | | |
| 3 | 1:80 (3) | 1:20 (1) | 1:80 (3) | 1:80 |
| 136 | 1:80 (3) | 1:20 (1) | 1:80 (3) | 1:80 |
| 169 | 1:160 (4) | 1:40 (2) | 1:160 (4) | 1:160 |
| 88 | 1:80 (3) | 1:20 (1) | 1:80 (3) | 1:80 |
| **IgG3** | | | | |
| 130 | 1:320 (5) | 1:80 (3) | 1:80 (3) | 1:80 |
| **IgG4** | | | | |
| 155 | 1:2560 (8) | 1:640 (6) | 1:1280 (7) | 1:1280 |
| 156 | 1:640 (6) | 1:80 (3) | 1:320 (5) | 1:320 |
| 23 | 1:160 (4) | 1:40 (2) | 1:80 (3) | 1:80 |
| 168 | 1:160 (4) | 1:20 (1) | 1:20 (1) | 1:20 |
| 55 | 1:80 (3) | 1:20 (1) | 1:40 (2) | 1:40 |
| 153 | 1:160 (4) | 1:20 (1) | 1:40 (2) | 1:40 |
| 143 | 1:80 (3) | 1:20 (1) | 1:40 (2) | 1:40 |
| *Abbreviations:*  ID = identity, refers to study code, IgG = immunoglobulin G. | | | | |

| **Table S4.** Characteristics of participants with non-neutralizing antibodies. | | | | | | | | | | | | |
| --- | --- | --- | --- | --- | --- | --- | --- | --- | --- | --- | --- | --- |
| **ID** | **Age (y)** | **Hemophilia severity** | **F*8* mutation** | **Treatment regimen** | **Cum. ED to FVIII** | **History of inhibitor development** | **Historical peak inhibitor titer (NBU/mL)** | **Received ITI** | **FVIII washout** | **Used product** | **FVIII-specific antibody** | **FVIII-specific antibody titer** |
| 1 | 21 | mild | unknown | on demand | >150 | never | n.a. | n.a. | n.a. | n.a. | IgG3 | 1:80 |
| 2 | 42 | mild | Missense  (p.Arg612Cys) | on demand | 101-150 | past | 0.5 | no | n.a. | n.a. | IgG1 and IgG4 | 1:160 and 1:1280 |
| 3 | 60 | mild | Missense  (p.Asn637Ser) | on demand | <50 | never | n.a. | n.a. | n.a. | n.a. | IgG1 | 1:20 |
| 4 | 75 | mild | Missense  (p.Arg612Cys) | prophylaxis | >150 | never | n.a. | n.a. | 3 days | SHL | IgM | 1:20 |
| 5 | 64 | mild | Missense  (p.Met633Ile) | on demand | <50^*^ | never | n.a. | n.a. | n.a. | n.a. | IgG1 | 1:20 |
| 6 | 42 | mild | Missense  (p.Arg612Cys) | on demand | <50^*^ | current | unknown | no | n.a. | n.a. | IgG1 and IgG4 | 1:320 and 1:5120 |
| 7 | 62 | mild | Missense  (p.Arg612Cys) | on demand | <50^*^ | never | n.a. | n.a. | n.a. | n.a. | IgG1 | 1:80 |
| 8 | 48 | mild | Missense  (p.Gln2248Arg) | on demand | >150 | never | n.a. | n.a. | n.a. | n.a. | IgG1 | 1:40 |
| 9 | 26 | mild | Missense  (p.Arg612Cys) | on demand | <50 | never | n.a. | n.a. | n.a. | n.a. | IgG1 and IgG3 | 1:40 and 1:20 |
| 10 | 64 | mild | Missense  (p.Arg612Cys) | on demand | 51-100 | never | n.a. | n.a. | n.a. | n.a. | IgG1 | 1:80 |
| 11 | 71 | mild | Missense (p.Arg612Cys) | on demand | <50^*^ | never | n.a. | n.a. | n.a. | n.a. | IgG1 | 1:40 |
| 12 | 57 | mild | Missense (p.Arg612Cys) | on demand | <50^*^ | past | 2.6 | no | n.a. | n.a. | IgG1 and IgG4 | 1:320 and 1:2560 |
| 13 | 50 | mild | unknown | on demand | <50 | never | n.a. | n.a. | n.a. | n.a. | IgA | 1:20 |
| 14 | 49 | mild | unknown | on demand | <50^*^ | never | n.a. | n.a. | n.a. | n.a. | IgG1 | 1:40 |
| 15 | 29 | mild | Missense (p.Tyr530His) | on demand | <50 | never | n.a. | n.a. | n.a. | n.a. | IgG1 | 1:40 |
| 16 | 17 | mild | Missense  (p.Arg2178Cys) | on demand | <50 | never | n.a. | n.a. | n.a. | n.a. | IgG3 | 1:40 |
| 17 | 8 | mild | Missense (p.Asp1260Glu) | on demand | <50 | never | n.a. | n.a. | n.a. | n.a. | IgA | 1:40 |
| 18 | 10 | mild | Missense (p.Asn637Ser) | on demand | <50 | never | n.a. | n.a. | n.a. | n.a. | IgG1 | 1:20 |
| 19 | 12 | mild | Missense (p.Arg612Cys) | on demand | <50 | never | n.a. | n.a. | n.a. | n.a. | IgG3 | 1:80 |
| 20 | 43 | mild | Missense (p.Arg612Cys) | on demand | <50 | never | n.a. | n.a. | n.a. | n.a. | IgG1 | 1:20 |
| 21 | 72 | mild | Missense (p.Arg612Cys) | on demand | 51-100 | never | n.a. | n.a. | n.a. | n.a. | IgG1 | 1:80 |
| 22 | 39 | mild | Missense (p.Ala200Thr) | on demand | 51-100 | never | n.a. | n.a. | n.a. | n.a. | IgG1 and IgM | 1:20 and 1:40 |
| 23 | 50 | mild | Missense (p.Arg612Cys) | on demand | <50 | current | 2.8 | no | n.a. | n.a. | IgG1 and IgG4 | 1:40 and 1:80 |
| 24 | 54 | mild | Missense (p.Thr55Met) | on demand | <50 | never | n.a. | n.a. | n.a. | n.a. | IgG1 | 1:80 |
| 25 | 17 | mild | Missense (p.1985Gln) | on demand | <50 | never | n.a. | n.a. | n.a. | n.a. | IgG1 | 1:40 |
| 26 | 19 | mild | unknown | on demand | <50 | never | n.a. | n.a. | n.a. | n.a. | IgM | 1:20 |
| 27 | 82 | mild | Missense (p.Leu1758Phe) | on demand | >150 | never | n.a. | n.a. | n.a. | n.a. | IgG3 | 1:80 |
| 28 | 55 | mild | unknown | on demand | 51-100 | never | n.a. | n.a. | n.a. | n.a. | IgG3 | 1:160 |
| 29 | 55 | mild | unknown | on demand | <50 | never | n.a. | n.a. | n.a. | n.a. | IgG1 | 1:20 |
| 30 | 46 | mild | Missense (p.Asn637Ser) | on demand | <50 | never | n.a. | n.a. | n.a. | n.a. | IgG3 | 1:20 |
| 31 | 50 | mild | Missense (p.Glu113Asp) | on demand | <50 | never | n.a. | n.a. | n.a. | n.a. | IgG1 | 1:40 |
| 32 | 84 | mild | Missense (p.Arg612Cys) | on demand | <50 | never | n.a. | n.a. | n.a. | n.a. | IgG3 | 1:20 |
| 33 | 67 | mild | Missense (p.Arg612Cys) | on demand | >150 | past | 3.3 | no | n.a. | n.a. | IgG1, IgG3, IgG4 and IgM | 1:640, 1:80, 1:160 and 1:40 |
| 34 | 29 | mild | Missense (p.Arg550Cys) | on demand | >150 | never | n.a. | n.a. | n.a. | n.a. | IgG1 | 1:40 |
| 35 | 51 | mild | Missense (p.Asp167Glu) | on demand | <50 | never | n.a. | n.a. | n.a. | n.a. | IgA | 1:40 |
| 36 | 20 | mild | unknown | on demand | <50 | never | n.a. | n.a. | n.a. | n.a. | IgG3 | 1:160 |
| 37 | 56 | mild | unknown | on demand | 51-100 | never | n.a. | n.a. | n.a. | n.a. | IgG1 | 1:20 |
| 38 | 37 | mild | unknown | on demand | >150 | never | n.a. | n.a. | n.a. | n.a. | IgG3 | 1:160 |
| 39 | 26 | mild | unknown | on demand | >150 | current | 0.7 | no | n.a. | n.a. | IgG1 | 1:80 |
| 40 | 18 | mild | Missense (p.Ser289Leu) | on demand | <50 | never | n.a. | n.a. | n.a. | n.a. | IgA | 1:160 |
| 41 | 53 | mild | unknown | on demand | 51-100 | never | n.a. | n.a. | n.a. | n.a. | IgG3 | 1:160 |
| 42 | 62 | mild | unknown | on demand | <50 | never | n.a. | n.a. | n.a. | n.a. | IgG1 and IgG3 | 1:40 and 1:40 |
| 43 | 69 | mild | No mutation in F*8* | on demand | <50 | never | n.a. | n.a. | n.a. | n.a. | IgG1 | 1:80 |
| 44 | 34 | mild | Intron 22 inversion | on demand | <50 | never | n.a. | n.a. | n.a. | n.a. | IgG3 | 1:20 |
| 45 | 56 | mild | Missense (p.Asp2307Val) | on demand | <50^*^ | never | n.a. | n.a. | n.a. | n.a. | IgG1 | 1:20 |
| 46 | 58 | mild | Missense (p.Arg2169His) | on demand | <50^*^ | never | n.a. | n.a. | n.a. | n.a. | IgG3 | 1:160 |
| 47 | 77 | mild | unknown | on demand | <50 | never | n.a. | n.a. | n.a. | n.a. | IgG3 | 1:40 |
| 48 | 72 | mild | unknown | on demand | <50 | never | n.a. | n.a. | n.a. | n.a. | IgG1 | 1:40 |
| 49 | 69 | mild | unknown | on demand | <50 | never | n.a. | n.a. | n.a. | n.a. | IgG1 | 1:40 |
| 50 | 62 | mild | unknown | on demand | 51-100 | past | 0.3 | no | n.a. | n.a. | IgG1, IgG3 and IgG4 | 1:80, 1:40 and 1:80 |
| 51 | 59 | mild | unknown | on demand | >150 | never | n.a. | n.a. | n.a. | n.a. | IgA | 1:160 |
| 52 | 71 | mild | unknown | on demand | 51-100 | never | n.a. | n.a. | n.a. | n.a. | IgG3 | 1:20 |
| 53 | 63 | mild | Missense (p.Asn637Ser) | on demand | <50 | never | n.a. | n.a. | n.a. | n.a. | IgG3 | 1:80 |
| 54 | 70 | mild | Missense (p.Arg717Leu) (p.Asp1260Glu) | on demand | <50^*^ | never | n.a. | n.a. | n.a. | n.a. | IgG1 | 1:20 |
| 55 | 54 | mild | Spontaneous mutation | on demand | <50^*^ | past | 4.0 | yes | n.a. | n.a. | IgG1 and IgG4 | 1:40 and 1:40 |
| 56 | 58 | mild | Missense (p.Val181Met) | on demand | <50 | never | n.a. | n.a. | n.a. | n.a. | IgG3 | 1:160 |
| 57 | 15 | mild | unknown | on demand | <50^*^ | never | n.a. | n.a. | n.a. | n.a. | IgG1 | 1:20 |
| 58 | 58 | mild | Missense (p.His228Tyr) | on demand | <50 | never | n.a. | n.a. | n.a. | n.a. | IgG3 and IgA | 1:40 and 1:40 |
| 59 | 72 | mild | Missense (p.Asn637Ser) | on demand | <50 | never | n.a. | n.a. | n.a. | n.a. | IgG1 | 1:160 |
| 60 | 62 | mild | unknown | prophylaxis | >150 | never | n.a. | n.a. | unknown | SHL | IgM | 1:40 |
| 61 | 34 | mild | unknown | on demand | <50 | never | n.a. | n.a. | n.a. | n.a. | IgM | 1:20 |
| 62 | 69 | mild | Missense (p.Arg612Cys) | on demand | <50 | never | n.a. | n.a. | n.a. | n.a. | IgG3 | 1:20 |
| 63 | 33 | mild | unknown | on demand | <50 | never | n.a. | n.a. | n.a. | n.a. | IgA | 1:40 |
| 64 | 48 | mild | Missense (p.Arg612Cys) | on demand | <50 | never | n.a. | n.a. | n.a. | n.a. | IgG3 | 1:20 |
| 65 | 30 | moderate | Missense (p.Arg2169His) | on demand | 50-150^*^ | never | n.a. | n.a. | n.a. |  | IgG1 | 1:20 |
| 66 | 63 | moderate | unknown | on demand | >150 | never | n.a. | n.a. | n.a. | n.a. | IgA | 1:40 |
| 67 | 3 | moderate | Missense (p.Val502Gly) | on demand | <50 | never | n.a. | n.a. | 2 days | SHL | IgG1 | 1:80 |
| 68 | 23 | moderate | Missense (p.Arg1708Cys) | prophylaxis | >150 | never | n.a. | n.a. | 3 days | SHL | IgG1 | 1:80 |
| 69 | 26 | moderate | unknown | prophylaxis | >150 | past | 76.0 | yes | 3 days | SHL | IgG1 and IgG3 | 1:80 and 1:20 |
| 70 | 63 | moderate | Missense (p.Arg1800His) | on demand | >150 | past | 8.3 | yes | n.a. | n.a. | IgG1 | 1:40 |
| 71 | 86 | moderate | Missense (p.Glu390Gly) | on demand | 51-100 | never | n.a. | n.a. | 3 days | SHL | IgG1 | 1:40 |
| 72 | 58 | moderate | Missense (p.Tyr431Asn) | on demand | <50 | never | n.a. | n.a. | n.a. | n.a. | IgA | 1:40 |
| 73 | 29 | moderate | unknown | on demand | <50 | never | n.a. | n.a. | n.a. | n.a. | IgG1 | 1:20 |
| 74 | 28 | moderate | Missense (p.Arg178His) | on demand | <50 | never | n.a. | n.a. | n.a. | n.a. | IgG1 | 1:40 |
| 75 | 28 | moderate | Missense (p.Ile2262Thr) | on demand | >150 | never | n.a. | n.a. | n.a. | n.a. | IgG3 | 1:20 |
| 76 | 27 | moderate | Missense (p.Arg2169His) | on demand | 50-150^*^ | past | 12.3 | no | n.a. | n.a. | IgG3 | 1:20 |
| 77 | 53 | moderate | Missense (p.Arg2169His) | prophylaxis | >150 | never | n.a. | n.a. | 1 day | SHL | IgG1 | 1:160 |
| 78 | 63 | moderate | unknown | on demand | 51-100 | never | n.a. | n.a. | n.a. | n.a. | IgG3 | 1:20 |
| 79 | 64 | moderate | Missense (p.Arg2169His) | on demand | >150 | never | n.a. | n.a. | n.a. | n.a. | IgG3 | 1:40 |
| 80 | 40 | moderate | Missense (p.Arg612Cys) | on demand | <50 | never | n.a. | n.a. | n.a. | n.a. | IgG3 and IgA | 1:20 and 1:40 |
| 81 | 7 | moderate | Missense (p.Arg2169His) | on demand | <50 | never | n.a. | n.a. | n.a. | n.a. | IgG3 | 1:40 |
| 82 | 55 | moderate | unknown | on demand | <50 | never | n.a. | n.a. | n.a. | n.a. | IgA | 1:40 |
| 83 | 23 | moderate | Missense (p.Gln2246Arg) | on demand | <50 | never | n.a. | n.a. | n.a. | n.a. | IgG3 | 1:20 |
| 84 | 74 | moderate | unknown | on demand | 51-100 | past | unknown | no | n.a. | n.a. | IgM | 1:160 |
| 85 | 47 | moderate | unknown | on demand | 51-100 | never | n.a. | n.a. | n.a. | n.a. | IgG3 | 1:40 |
| 86 | 39 | moderate | unknown | on demand | 101-150 | never | n.a. | n.a. | n.a. | n.a. | IgG1 | 1:20 |
| 87 | 32 | moderate | unknown | prophylaxis | >150 | never | n.a. | n.a. | 3 days | SHL | IgG3 | 1:40 |
| 88 | 28 | moderate | Missense  c.6794G>A | on demand | >150 | never | n.a. | n.a. | n.a. | n.a. | IgG1 | 1:80 |
| 89 | 71 | moderate | Missense c.1689C>T (Arg>Cys) | prophylaxis | >150 | never | n.a. | n.a. | 3 days | SHL | IgG3 | 1:40 |
| 90 | 43 | severe | Intron 22 inversion | on demand | >150 | past | 0.7 | no | n.a. | n.a. | IgG1 and IgA | 1:40 and 1:80 |
| 91 | 45 | severe | Intron 22 inversion | prophylaxis | >150 | never | n.a. | n.a. | 2 days | SHL | IgG1 | 1:20 |
| 92 | 33 | severe | Nonsense (p.Ser1176Stop) | prophylaxis | >150 | past | 0.5 | no | 0 days | SHL | IgA | 1:20 |
| 93 | 5 | severe | Intron 22 inversion | prophylaxis | >150 | past | 3.2 | yes | 1 day | SHL | IgA | 1:20 |
| 94 | 12 | severe | Intron 22 inversion | prophylaxis | >150 | never | n.a. | n.a. | 1 day | SHL | IgA | 1:20 |
| 95 | 6 | severe | Intron 22 inversion | prophylaxis | >150^*^ | current | 300 | yes | unknown | SHL | IgG4 | 1:1280 |
| 96 | 12 | severe | Missense (p.Asp1260Glu) | prophylaxis | >150 | never | n.a. | n.a. | >3 days | SHL | IgG1 | 1:80 |
| 97 | 23 | severe | Intron 22 inversion | prophylaxis | >150 | never | n.a. | n.a. | 3 days | SHL | IgG1 | 1:20 |
| 98 | 35 | severe | unknown | prophylaxis | >150 | never | n.a. | n.a. | 0 days | SHL | IgA | 1:160 |
| 99 | 7 | severe | Intron 22 inversion | prophylaxis | >150 | past | 224.0 | yes | unknown | SHL | IgG4 | 1:40 |
| 100 | 12 | severe | unknown | prophylaxis | >150 | never | n.a. | n.a. | 2 days | SHL | IgG1 | 1:40 |
| 101 | 10 | severe | unknown | prophylaxis | >150 | never | n.a. | n.a. | 2 days | SHL | IgG1 and IgM | 1:40 and 1:40 |
| 102 | 7 | severe | unknown | prophylaxis | >150 | never | n.a. | n.a. | 1 day | EHL | IgG1 | 1:20 |
| 103 | 2 | severe | unknown | prophylaxis | >150 | past | 1.1 | yes | 2 days | EHL | IgG1 and IgG4 | 1:40 and 1:1280 |
| 104 | 10 | severe | Splice site (IVS16-1G>T) | prophylaxis | >150 | past | 3.2 | yes | 3 days | EHL | IgG3 | 1:40 |
| 105 | 14 | severe | Intron 22 inversion | prophylaxis | >150 | never | n.a. | n.a. | >3 days | EHL | IgG1 | 1:40 |
| 106 | 42 | severe | Missense (p.Ser1839Cys) | prophylaxis | >150 | never | n.a. | n.a. | >3 days | EHL | IgG3 | 1:320 |
| 107 | 47 | severe | Large deletion exon 11 | prophylaxis | >150 | never | n.a. | n.a. | 0 days | SHL | IgA | 1:20 |
| 108 | 48 | severe | Intron 22 inversion + missense (p.Asp1241Gln) | prophylaxis | >150 | never | n.a. | n.a. | 1 day | SHL | IgG1 | 1:40 |
| 109 | 30 | severe | Intron 22 inversion | prophylaxis | >150 | never | n.a. | n.a. | 3 days | SHL | IgG3 | 1:20 |
| 110 | 58 | severe | unknown | on demand | >150 | never | n.a. | n.a. | 0 days | SHL | IgG1 and IgG3 | 1:80 and 1:640 |
| 111 | 58 | severe | Intron 22 inversion | prophylaxis | >150 | never | n.a. | n.a. | 0 days | EHL | IgG1 | 1:20 |
| 112 | 21 | severe | Splice site  IVS14+2(T>C) | on demand | >150 | never | n.a. | n.a. | 0 days | SHL | IgG3 | 1:20 |
| 113 | 82 | severe | Intron 22 inversion | prophylaxis | >150 | never | n.a. | n.a. | 2 days | SHL | IgG3 | 1:80 |
| 114 | 56 | severe | Missense (p.Pro2300Arg) | on demand | >150 | never | n.a. | n.a. | n.a. | n.a. | IgG1 | 1:20 |
| 115 | 54 | severe | unknown | on demand | >150 | never | n.a. | n.a. | n.a. | n.a. | IgG1 | 1:80 |
| 116 | 33 | severe | Small insertion (5962insA) | prophylaxis | >150 | never | n.a. | n.a. | 1 day | EHL | IgG1 | 1:20 |
| 117 | 19 | severe | unknown | prophylaxis | >150 | never | n.a. | n.a. | 2 days | SHL | IgG3 | 1:80 |
| 118 | 54 | severe | unknown | prophylaxis | >150 | never | n.a. | n.a. | 2 days | SHL | IgA | 1:40 |
| 119 | 37 | severe | Missense (p.Arg2163His) | prophylaxis | >150 | never | n.a. | n.a. | 2 days | SHL | IgG1 | 1:20 |
| 120 | 41 | severe | unknown | prophylaxis | >150 | never | n.a. | n.a. | 1 day | EHL | IgG3 | 1:20 |
| 121 | 48 | severe | Intron 22 inversion | prophylaxis | >150 | never | n.a. | n.a. | 1 day | SHL | IgG1 | 1:20 |
| 122 | 65 | severe | Intron 22 inversion | on demand | >150 | never | n.a. | n.a. | 1 day | SHL | IgG1 | 1:20 |
| 123 | 47 | severe | Missense (p.Glu79Lys) | on demand | >150 | never | n.a. | n.a. | 3 days | SHL | IgG3 | 1:20 |
| 124 | 75 | severe | Intron 22 inversion | prophylaxis | >150 | never | n.a. | n.a. | 1 day | EHL | IgG1 | 1:160 |
| 125 | 23 | severe | unknown | prophylaxis | >150 | never | n.a. | n.a. | >3 days | SHL | IgG1 | 1:20 |
| 126 | 68 | severe | Small deletion (209delITTGT) | prophylaxis | >150 | never | n.a. | n.a. | 0 days | SHL | IgG1 | 1:20 |
| 127 | 44 | severe | Small insertion (1966-1969InsA) | on demand | >150 | never | n.a. | n.a. | n.a. | n.a. | IgA | 1:40 |
| 128 | 64 | severe | unknown | prophylaxis | >150 | never | n.a. | n.a. | 1 day | SHL | IgA | 1:40 |
| 129 | 21 | severe | unknown | prophylaxis | >150 | never | n.a. | n.a. | 0 days | SHL | IgG1 | 1:80 |
| 130 | 26 | severe | unknown | prophylaxis | >150 | never | n.a. | n.a. | 1 day | SHL | IgG3 | 1:80 |
| 131 | 30 | severe | Small insertion (6321insAC) | prophylaxis | >150 | never | n.a. | n.a. | 1 day | EHL | IgG1 | 1:80 |
| 132 | 49 | severe | Intron 22 inversion | prophylaxis | >150 | never | n.a. | n.a. | 0 days | SHL | IgG1 | 1:20 |
| 133 | 7 | severe | Missense c.5219+1G>A | prophylaxis | >150 | never | n.a. | n.a. | 1 day | SHL | IgG1 | 1:20 |
| 134 | 51 | severe | Intron 22 inversion | prophylaxis | >150 | never | n.a. | n.a. | 1 day | EHL | IgG1 | 1:20 |
| 135 | 18 | severe | unknown | prophylaxis | >150 | never | n.a. | n.a. | unknown | SHL | IgG3 | 1:40 |
| 136 | 56 | severe | Missense (p.Arg48Lys) | prophylaxis | >150 | never | n.a. | n.a. | 2 days | SHL | IgG1 | 1:80 |
| 137 | 53 | severe | unknown | prophylaxis | >150 | past | 3.0 | no | >3 days | EHL | IgG4 | 1:40 |
| 138 | 24 | severe | Small insertion (2150InsA) | prophylaxis | >150 | never | n.a. | n.a. | 2 days | SHL | IgG3 | 1:20 |
| 139 | 65 | severe | unknown | prophylaxis | >150 | never | n.a. | n.a. | >3 days | SHL | IgG1 | 1:20 |
| 140 | 24 | severe | Intron 22 inversion | prophylaxis | >150 | never | n.a. | n.a. | unknown | SHL | IgG1 | 1:20 |
| 141 | 31 | severe | Intron 22 inversion | prophylaxis | >150 | never | n.a. | n.a. | 2 days | SHL | IgG1 | 1:40 |
| 142 | 63 | severe | Intron 22 inversion | prophylaxis | >150 | never | n.a. | n.a. | 1 day | SHL | IgM | 1:20 |
| 143 | 13 | severe | Intron 22 inversion | prophylaxis | >150 | past | 48.0 | yes | unknown | SHL | IgG4 | 1:40 |
| 144 | 74 | severe | Splice site  IVS20-1(G>C) | prophylaxis | >150 | never | n.a. | n.a. | 3 days | SHL | IgG1 and IgA | 1:20 and 1:20 |
| *Abbreviations*. ID = identity, refers to study code, y = years, ED = exposure days, ITI = immune tolerance induction, n.a. = not applicable, SHL = standard half-life, EHL = extended half-life, IgM = immunoglobulin M, IgG = immunoglobulin G. ^†^ Washout period was mentioned for persons receiving prophylaxis and for persons treated on demand with a recent bleed for which they were treated with FVIII concentrates. ^*^ imputed data. | | | | | | | | | | | | |

| **Table S5.** Overview of antibody isotypes and subclasses for NNA | |
| --- | --- |
| **Antibody subclass and isotype** | **N** |
| **Only IgG antibodies** | **113** |
| IgG1 | 61 |
| IgG3 | 38 |
| IgG4 | 3 |
| IgG1 + IgG3 | 4 |
| IgG1 + IgG4 | 6 |
| IgG1 + IgG3 + IgG4 | 1 |
|  |  |
| **Only IgM antibodies** | **6** |
|  |  |
| **Only IgA antibodies** | **17** |
|  |  |
| **Both IgG and IgM antibodies** | **3** |
| IgG1 + IgM | 2 |
| IgG1 + IgG3 + IgG4 + IgM | 1 |
|  |  |
| **Both** **IgG and IgA antibodies** | **4** |
| IgG1 + IgA | 2 |
| IgG3 + IgA | 2 |

| **Table S6.** Prevalence of non-neutralizing and neutralizing antibodies per different mutation type (null or non-null mutations). | | | | |
| --- | --- | --- | --- | --- |
|  | **Total (N=788)** | **Non-neutralizing antibodies**  **(N = 143)** | **Neutralizing antibodies**  **(N = 23)** | ***p-value^*^*** |
| Non-null mutations | 356 | 73 (20.5) | 8 (2.2) | 0.098 |
| Null mutations | 134 | 21 (15.7) | 6 (4.5) |  |
| No mutation | 3 | 1 (33.3) | 1 (33.3) |  |
| *Not determined* | *295* | *48 (16.3)* | *8 (2.7)* |  |
| * Pearson Chi-Square shows no statistical difference between the prevalence of neutralizing or non-neutralizing antibodies between persons with null or non-null *F8* gene mutations. For non-neutralizing antibodies, non-null mutations included missense mutation (n=64), small deletion or insertion (n=5), and splice site mutation (n=3), and a small spontaneous mutation (n=1), and null mutations included intron 22 inversion (n=21). For neutralizing antibodies, non-null mutations included missense mutation (n=7), small deletion or insertion (n=1), and null-mutations included intron 22 inversion (n=3), nonsense mutation (n=2), and large deletion (n=1). | | | | |

| **Table S7.** Characteristics of participants with very low-titer inhibitors. | | | | | | | | | | | | | | |
| --- | --- | --- | --- | --- | --- | --- | --- | --- | --- | --- | --- | --- | --- | --- |
| **ID** | **Age (y)** | **Hemophilia severity** | **F*8* mutation** | **Treatment regimen** | **Cum.**  **ED to**  **FVIII** | **History of inhibitor development** | **Historical peak inhibitor titer (NBU/mL)** | **Received ITI** | **FVIII washout^†^** | **Used product** | **FVIII-specific antibody** | **FVIII-specific antibody titer** | **NusBA**  **(NusBU/mL)** | **NBA (NBU/mL)** |
| 145 | 41 | mild | Missense (p.Hys161Tyr) | on demand | <50 | never | n.a. | n.a. | n.a. | n.a. | - | - | 0.13 | - |
| 146 | 42 | mild | unknown | on demand | <50 | past or current | unknown | unknown | n.a. | n.a. | IgA | 1:40 | 0.46 | - |
| 147 | 75 | mild | unknown | on demand | >150 | never | n.a. | n.a. | n.a. | n.a. | - | - | 0.18 | - |
| 148 | 23 | moderate | Missense (p.Ala2218Thr) | on demand | >150 | never | n.a. | n.a. | 2 days | SHL | IgG1 | 1:40 | 0.11 | - |
| 149 | 50 | moderate | Missense (p.Arg2169His) | on demand | <50 | past or current | 30.9 | no | n.a. | n.a. | IgG1 and IgG4 | 1:80 and 1:160 | >0.8 | - |
| 150 | 66 | severe | unknown | on demand | >150 | past | 25.0 | yes | n.a. | n.a. | IgG1 and IgG4 | 1:20 and  1:40 | 0.16 | - |
| 151 | 2 | severe | Intron 22 inversion | prophylaxis | >150 | past | 73.6 | yes | 1 day | SHL | IgG4 | 1:80 | 0.22 | - |
| 152 | 14 | severe | Intron 22 inversion | prophylaxis | <50 | current | 200 | yes | >3 days | Emicizumab | IgG1 | 1:40 | 0.44 | - |
| 153 | 4 | severe | Missense (p.Cys12) | prophylaxis | >150 | never | n.a. | n.a. | 3 days | SHL | IgG4 | 1:40 | 0.20 | - |
| 154 | 59 | severe | Nonsense (p.Arg1696Stop) | prophylaxis | >150 | past | 19.2 | yes | unknown | SHL | - | - | 0.12 | - |
| *Abbreviations.* ID = identity, refers to study code, y = years, ITI = immune tolerance induction, n.a. = not applicable, SHL = standard half-life, - = negative, + = positive, IgG = immunoglobulin G, NusBA = Nijmegen ultra-sensitive Bethesda assay, NBA = Nijmegen Bethesda assay, NusBU = Nijmegen ultra-sensitive Bethesda units, NBU = Nijmegen Bethesda units. ^†^ Washout period was mentioned for persons receiving prophylaxis and for persons treated on demand with a recent bleed for which they were treated with FVIII concentrates. | | | | | | | | | | | | | | |

| **Table S8.** Characteristics of participants with positive results in one or two assays, but antibodies were unclassified due to other missing data | | | | | | | | | | | | | | | | | | | | | | |
| --- | --- | --- | --- | --- | --- | --- | --- | --- | --- | --- | --- | --- | --- | --- | --- | --- | --- | --- | --- | --- | --- | --- |
| **ID** | **Age (y)** | **Hemophilia severity** | **F*8* mutation** | **Treatment regimen** | **Cum.**  **ED to**  **FVIII** | **History of inhibitor development** | **Historical peak inhibitor titer (NBU/mL)** | | **Received ITI** | | **FVIII washout^†^** | | **Used product** | | **FVIII-specific antibody** | | **FVIII-specific antibody titer** | | **NusBA (NusBU/mL)** | | | **NBA**  **(NBU/mL)** |
| 168 | 35 | moderate | Missense (p.Arg2169His) | on demand | 50-150^*^ | current | 13.2 | | no | | n.a. | | n.a. | | IgG1 and IgG4 | | 1:20 and 1:20 | | missing | | | Depending on NusBA results |
| 169 | 7 | moderate | unknown | on demand | <50 | never | n.a. | n.a. | | n.a. | | n.a. | | IgG1 and IgG3 | | 1:160 and 1:40 | | missing | | Depending on NusBA results |  |  |
| 170 | 3 | severe | unknown | prophylaxis | >150 | current | 1200 | yes | | 2 days | | SHL | | IgG1,  IgG2, IgG3 and IgG4 | | 1:5120, 1:320, 1:140 and 1:40960 | | missing | | Depending on NusBA results |  |  |
| *Abbreviations.* ID = identity, refers to study code, y = years, ED = exposure days, ITI = immune tolerance induction, n.a. = not applicable, SHL = standard half-life, - = negative, + = positive, IgG = immunoglobulin G, NusBA = Nijmegen ultra-sensitive Bethesda assay, NBA = Nijmegen Bethesda assay, NusBU = Nijmegen ultra-sensitive Bethesda units, NBU = Nijmegen Bethesda units. ^†^ Washout period was mentioned for persons receiving prophylaxis and for persons treated on demand with a recent bleed for which they were treated with FVIII concentrates. ^*^ imputed data. | | | | | | | | | | | | | | | | | | | | |  |  |

| **Table S9.** Characteristics of participants with inhibitors. | | | | | | | | | | | | | | | | |
| --- | --- | --- | --- | --- | --- | --- | --- | --- | --- | --- | --- | --- | --- | --- | --- | --- |
| **ID** | **Age (y)** | **Hemophilia severity** | **F*8* mutation** | **Treatment regimen** | **Cum.**  **ED to**  **FVIII** | **History of inhibitor development** | **Historical peak inhibitor titer (NBU/mL)** | **Received ITI** | **FVIII washout^†^** | | **Used product** | **FVIII-specific antibody** | **FVIII-specific antibody titer** | **NusBA (NusBU/ mL)** | **NBA (NBU/ mL)** | **Anti phospolipid antibodies^†^** |
| 155 | 25 | mild | Missense (p.Arg612Cys) | on demand | <50 | never | n.a. | n.a. | n.a. | n.a. | | IgG1 and IgG4 | 1:160 and 1:1280 | 0.11 | 0.71 | n.a. |
| 156 | 72 | mild | unknown | on demand | 51-100 | current | 5.0 | no | n.a. | | n.a. | IgG1 and IgG4 | 1:80 and 1:320 | 0.12 | 0.90 | n.a. |
| 157 | 62 | mild | Missense (p.Arg612Cys) | on demand | >150 | current | 2.1 | no | n.a. | | n.a. | IgG1 and IgG4 | 1:320 and 1:20480 | 0.49 | 1.36 | n.a. |
| 158 | 50 | mild | No mutation in F*8* | on demand | >150 | past | 12.3 | no | n.a. | | n.a. | - | - | >0.8 | 2.80 | - |
| 159 | 66 | moderate | unknown | on demand | >150 | current | 307 | no | n.a. | | n.a. | IgG1 and IgG4 | 1:640 and 1:640 | 0.20 | 1.36 | n.a. |
| 160 | 74 | moderate | unknown | on demand | >150 | past | 4.3 | yes | n.a. | | n.a. | IgG1 and IgA | 1:20 and 1:20 | >0.8 | 0.92 | n.a. |
| 161 | 4 | severe | unknown | prophylaxis | >150 | current | 72.8 | yes | 2 days | | aPCC | IgG1 and IgG4 | 1:40 and 1:10240 | >0.8 | missing^‡^ | n.a. |
| 162 | 46 | severe | Large deletion (exon 1-22) | on demand | >150 | current | 206 | no | n.a. | | n.a. | IgG1, IgG3 and IgG4 | 1:320, 1:160 and 1:2560 | >0.8 | 7.0 | n.a. |
| 163 | 34 | severe | Intron 22 inversion | prophylaxis | >150 | current | 1.0 | no | >3 days | | SHL | - | - | 0.33 | 1.0 | - |
| 164 | 48 | severe | Missense (p.Asp1241Glu) | prophylaxis | >150 | current | 900 | yes | >3 days | | Emicizumab, SHL | IgG1 and IgG4 | 1:320 and 1:10240 | >0.8 | 9 | n.a. |
| 165 | 47 | severe | unknown | prophylaxis | >150 | current | 900 | Yes | >3 days | | Emicizumab, SHL | IgG1, IgG2 and IgG4 | 1:640, 1:160 and 1:20480 | >0.8 | 47 | n.a. |
| 166 | 16 | severe | Nonsense (p.Arg1966X) | prophylaxis | <50 | current | 600 | Yes | >3 days | | Emicizumab | IgG1 and IgG4 | 1:160 and 1:640 | >0.8 | 4.5 | n.a. |
| 167 | 59 | severe | Small deletion (p.Tle613fs) | prophylaxis | >150 | past | 7.1 | yes | >3 days | | Emicizumab, SHL | IgG1 and IgG4 | 1:160 and 1:160 | >0.8 | 2.2 | n.a. |
| *Abbreviations.* ID = identity, refers to study code, y = years, ITI = immune tolerance induction, n.a. = not applicable, NP = not performed, SHL = standard half-life, aPCC = activated prothombin complex concentrates, - = negative, + = positive, IgG = immunoglobulin G, NusBA = Nijmegen ultra-sensitive Bethesda assay, NBA = Nijmegen Bethesda assay, NusBU = Nijmegen ultra-sensitive Bethesda units, NBU = Nijmegen Bethesda units. † Anti-cardiolipin and beta2-glycoprotein antibodies, only tested in two participants with discrepant results between ELISA and NBA. ^†^ Washout period was mentioned for persons receiving prophylaxis and for persons treated on demand with a recent bleed for which they were treated with FVIII concentrates. ^‡^ The NBA results were missing due insufficient material to perform this assay, nevertheless, the detected antibodies were classified as inhibitors given the high NusBA titer together with the high IgG4 antibody titers detected in ELISA. | | | | | | | | | | | | | | | | |

| **Table S10.** Overview of negative ELISA results in persons with positive NusBA results (very low-titer inhibitor) | | | | | | | | | | | |
| --- | --- | --- | --- | --- | --- | --- | --- | --- | --- | --- | --- |
| **ID** | **Antibody subclass or isotype** | **Detection ELISA** | | | |  | **Specificity ELISA** | | | | **Possible explanation discrepancy ELISA and NusBA/NBA** |
|  |  | **ELISA (OD)** | **NC mean (OD)** | **NC cutoff (OD)** | **Defined as** |  | **ELISA (OD) – without comp.** | **ELISA (OD) – with comp.** | **Signal reduction (%) with comp.** | **Defined as** |  |
| **145** | IgG1 | 0.094 | 0.048 | 0.095 | Negative |  |  |  |  |  | Results at lower detection limit of both IgG1 and IgG4 ELISA, and below the cut-off for FVIII-specificity for IgG4 subclass antibodies |
|  |  | 0.078 | 0.046 | 0.092 | Negative |  |  |  |  |  |  |
|  | IgG2 | 0.014 | 0.018 | 0.036 | Negative |  |  |  |  |  |  |
|  |  | 0.015 | 0.018 | 0.036 | Negative |  |  |  |  |  |  |
|  | IgG3 | 0.015 | 0.021 | 0.041 | Negative |  |  |  |  |  |  |
|  |  | 0.016 | 0.030 | 0.060 | Negative |  |  |  |  |  |  |
|  | IgG4 | 0.025 | 0.008 | 0.017 | Positive |  | 0.019  (OD < NC) | 0.020 | +5 |  |  |
|  |  | 0.018 | 0.011 | 0.021 | Negative |  | 0.018  (OD < NC) | 0.018 | 0 |  |  |
|  |  | 0.013 | 0.006 | 0.013 | Positive |  |  |  |  |  |  |
|  |  | 0.015 | 0.010 | 0.020 | Negative |  |  |  |  |  |  |
|  |  | 0.024 | 0.010 | 0.020 | Positive |  |  |  |  |  |  |
|  | IgM | 0.114 | 0.097 | 0.195 | Negative |  |  |  |  |  |  |
|  |  | 0.098 | 0.088 | 0.176 | Negative |  |  |  |  |  |  |
|  | IgA | 0.080 | 0.069 | 0.139 | Negative |  |  |  |  |  |  |
|  |  | 0.080 | 0.070 | 0.139 | Negative |  |  |  |  |  |  |
| **147** | IgG1 | 0.070 | 0.044 | 0.088 | Negative |  |  |  |  |  | Use of DOACs |
|  |  | 0.069 | 0.040 | 0.080 | Negative |  |  |  |  |  |  |
|  |  | 0.035 | 0.027 | 0.054 | Negative |  |  |  |  |  |  |
|  |  | 0.036 | 0.027 | 0.054 | Negative |  |  |  |  |  |  |
|  |  | 0.037 | 0.027 | 0.054 | Negative |  |  |  |  |  |  |
|  |  | 0.041 | 0.027 | 0.054 | Negative |  |  |  |  |  |  |
|  | IgG2 | 0.018 | 0.020 | 0.040 | Negative |  |  |  |  |  |  |
|  |  | 0.016 | 0.021 | 0.042 | Negative |  |  |  |  |  |  |
|  | IgG3 | 0.016 | 0.028 | 0.055 | Negative |  |  |  |  |  |  |
|  |  | 0.009 | 0.016 | 0.032 | Negative |  |  |  |  |  |  |
|  |  | 0.013 | 0.025 | 0.051 | Negative |  |  |  |  |  |  |
|  |  | 0.012 | 0.025 | 0.051 | Negative |  |  |  |  |  |  |
|  |  | 0.014 | 0.025 | 0.051 | Negative |  |  |  |  |  |  |
|  |  | 0.012 | 0.025 | 0.051 | Negative |  |  |  |  |  |  |
|  | IgG4 | 0.005 | 0.007 | 0.014 | Negative |  |  |  |  |  |  |
|  |  | 0.005 | 0.006 | 0.012 | Negative |  |  |  |  |  |  |
|  |  | 0.007 | 0.011 | 0.021 | Negative |  |  |  |  |  |  |
|  |  | 0.008 | 0.011 | 0.021 | Negative |  |  |  |  |  |  |
|  |  | 0.005 | 0.011 | 0.021 | Negative |  |  |  |  |  |  |
|  |  | 0.008 | 0.011 | 0.021 | Negative |  |  |  |  |  |  |
|  | IgM | 0.103 | 0.083 | 0.166 | Negative |  |  |  |  |  |  |
|  |  | 0.094 | 0.075 | 0.150 | Negative |  |  |  |  |  |  |
|  | IgA | 0.039 | 0.030 | 0.061 | Negative |  |  |  |  |  |  |
|  |  | 0.044 | 0.033 | 0.065 | Negative |  |  |  |  |  |  |
| **154** | IgG1 | 0.066 | 0.052 | 0.105 | Negative |  |  |  |  |  | Results at lower detection limit of IgG4 ELISA |
|  |  | 0.071 | 0.050 | 0.100 | Negative |  |  |  |  |  |  |
|  |  | 0.034 | 0.027 | 0.054 | Negative |  |  |  |  |  |  |
|  |  | 0.031 | 0.027 | 0.054 | Negative |  |  |  |  |  |  |
|  |  | 0.035 | 0.027 | 0.054 | Negative |  |  |  |  |  |  |
|  |  | 0.033 | 0.027 | 0.054 | Negative |  |  |  |  |  |  |
|  | IgG2 | 0.019 | 0.016 | 0.032 | Negative |  |  |  |  |  |  |
|  |  | 0.011 | 0.009 | 0.019 | Negative |  |  |  |  |  |  |
|  | IgG3 | 0.009 | 0.024 | 0.048 | Negative |  |  |  |  |  |  |
|  |  | 0.008 | 0.022 | 0.043 | Negative |  |  |  |  |  |  |
|  |  | 0.013 | 0.025 | 0.051 | Negative |  |  |  |  |  |  |
|  |  | 0.012 | 0.025 | 0.051 | Negative |  |  |  |  |  |  |
|  |  | 0.015 | 0.025 | 0.051 | Negative |  |  |  |  |  |  |
|  |  | 0.011 | 0.025 | 0.051 | Negative |  |  |  |  |  |  |
|  | IgG4 | 0.013 | 0.008 | 0.016 | Negative |  | 0.023 (OD < NC) | 0.005 | 78 | Specific |  |
|  |  | 0.013 | 0.006 | 0.011 | Positive |  | 0.035 (OD > NC) | 0.007 | 80 | Specific |  |
|  |  | 0.021 | 0.011 | 0.021 | Positive |  |  |  |  |  |  |
|  |  | 0.020 | 0.011 | 0.021 | Negative |  |  |  |  |  |  |
|  |  | 0.020 | 0.011 | 0.021 | Negative |  |  |  |  |  |  |
|  |  | 0.022 | 0.011 | 0.021 | Positive |  |  |  |  |  |  |
|  |  | 0.023 | 0.013 | 0.026 | Negative |  |  |  |  |  |  |
|  |  | 0.035 | 0.013 | 0.026 | Positive |  |  |  |  |  |  |
|  | IgM | 0.024 | 0.050 | 0.100 | Negative |  |  |  |  |  |  |
|  |  | 0.021 | 0.045 | 0.090 | Negative |  |  |  |  |  |  |
|  | IgA | 0.081 | 0.068 | 0.136 | Negative |  |  |  |  |  |  |
|  |  | 0.083 | 0.064 | 0.128 | Negative |  |  |  |  |  |  |
| *Àbbreviations.* OD = optical density, ELISA = enzyme-linked immunoassay, comp. = competition, NusBA = Nijmegen ultra-sensitive Bethesda assay, NBA = Nijmegen Bethesda assay, DOAC = direct oral anticoagulant, NC = negative control. | | | | | | | | | | | |

| **Table S11.** Overview of negative ELISA results in persons with positive NusBA results (very low-titer inhibitor) | | | | | | | | | | | |
| --- | --- | --- | --- | --- | --- | --- | --- | --- | --- | --- | --- |
| **ID** | **Antibody subclass or isotype** | **Detection ELISA** | | | |  | **Specificity ELISA** | | | | **Possible explanation discrepancy ELISA and NusBA/NBA** |
|  |  | **ELISA (OD)** | **NC mean (OD)** | **NC cutoff (OD)** | **Defined as** |  | **ELISA (OD) – without comp.** | **ELISA (OD) – with comp.** | **Signal reduction (%) with comp.** | **Defined as** |  |
| **158** | IgG1 | 0.070 | 0.035 | 0.069 | Positive |  |  |  |  |  | Results at lower detection limit of IgG1 ELISA and below the cut-off for FVIII-specificity for IgM subclass antibodies |
|  |  | 0.073 | 0.040 | 0.080 | Negative |  |  |  |  |  |  |
|  |  | 0.036 | 0.023 | 0.045 | Negative |  |  |  |  |  |  |
|  |  | 0.035 | 0.023 | 0.045 | Negative |  |  |  |  |  |  |
|  |  | 0.032 | 0.023 | 0.045 | Negative |  |  |  |  |  |  |
|  |  | 0.036 | 0.023 | 0.045 | Negative |  |  |  |  |  |  |
|  | IgG2 | 0.008 | 0.013 | 0.025 | Negative |  |  |  |  |  |  |
|  |  | 0.008 | 0.014 | 0.028 | Negative |  |  |  |  |  |  |
|  | IgG3 | 0.006 | 0.015 | 0.030 | Negative |  |  |  |  |  |  |
|  |  | 0.006 | 0.019 | 0.038 | Negative |  |  |  |  |  |  |
|  |  | 0.016 | 0.027 | 0.054 | Negative |  |  |  |  |  |  |
|  |  | 0.011 | 0.027 | 0.054 | Negative |  |  |  |  |  |  |
|  |  | 0.019 | 0.027 | 0.054 | Negative |  |  |  |  |  |  |
|  |  | 0.007 | 0.027 | 0.054 | Negative |  |  |  |  |  |  |
|  | IgG4 | 0.005 | 0.008 | 0.015 | Negative |  |  |  |  |  |  |
|  |  | 0.004 | 0.009 | 0.017 | Negative |  |  |  |  |  |  |
|  |  | 0.008 | 0.013 | 0.026 | Negative |  |  |  |  |  |  |
|  |  | 0.007 | 0.013 | 0.026 | Negative |  |  |  |  |  |  |
|  |  | 0.011 | 0.013 | 0.026 | Negative |  |  |  |  |  |  |
|  |  | 0.016 | 0.013 | 0.026 | Negative |  |  |  |  |  |  |
|  | IgM | 0.165 | 0.056 | 0.113 | Positive |  | 0.162 | 0.106 | 35 | Unspecific |  |
|  |  | 0.161 | 0.056 | 0.112 | Positive |  | 0.153 | 0.107 | 30 | Unspecific |  |
|  | IgA | 0.042 | 0.037 | 0.074 | Negative |  |  |  |  |  |  |
|  |  | 0.046 | 0.041 | 0.082 | Negative |  |  |  |  |  |  |
| **163** | IgG1 | 0.026 | 0.035 | 0.069 | Negative |  |  |  |  |  | Unexplained |
|  |  | 0.028 | 0.040 | 0.080 | Negative |  |  |  |  |  |  |
|  |  | 0.017 | 0.027 | 0.054 | Negative |  |  |  |  |  |  |
|  |  | 0.016 | 0.027 | 0.054 | Negative |  |  |  |  |  |  |
|  |  | 0.018 | 0.027 | 0.054 | Negative |  |  |  |  |  |  |
|  |  | 0.017 | 0.027 | 0.054 | Negative |  |  |  |  |  |  |
|  | IgG2 | 0.010 | 0.013 | 0.025 | Negative |  |  |  |  |  |  |
|  |  | 0.017 | 0.014 | 0.028 | Negative |  |  |  |  |  |  |
|  | IgG3 | 0.007 | 0.015 | 0.030 | Negative |  |  |  |  |  |  |
|  |  | 0.006 | 0.019 | 0.038 | Negative |  |  |  |  |  |  |
|  |  | 0.010 | 0.025 | 0.051 | Negative |  |  |  |  |  |  |
|  |  | 0.009 | 0.025 | 0.051 | Negative |  |  |  |  |  |  |
|  |  | 0.009 | 0.025 | 0.051 | Negative |  |  |  |  |  |  |
|  |  | 0.010 | 0.025 | 0.051 | Negative |  |  |  |  |  |  |
|  | IgG4 | 0.005 | 0.008 | 0.015 | Negative |  |  |  |  |  |  |
|  |  | 0.005 | 0.009 | 0.017 | Negative |  |  |  |  |  |  |
|  |  | 0.007 | 0.011 | 0.021 | Negative |  |  |  |  |  |  |
|  |  | 0.006 | 0.011 | 0.021 | Negative |  |  |  |  |  |  |
|  |  | 0.006 | 0.011 | 0.021 | Negative |  |  |  |  |  |  |
|  |  | 0.008 | 0.011 | 0.021 | Negative |  |  |  |  |  |  |
|  | IgM | 0.087 | 0.056 | 0.113 | Negative |  |  |  |  |  |  |
|  |  | 0.082 | 0.056 | 0.112 | Negative |  |  |  |  |  |  |
|  | IgA | 0.035 | 0.037 | 0.074 | Negative |  |  |  |  |  |  |
|  |  | 0.040 | 0.041 | 0.082 | Negative |  |  |  |  |  |  |
| *Abbreviations.* OD = optical density, ELISA = enzyme-linked immunoassay, comp. = competition, NusBA = Nijmegen ultra-sensitive Bethesda assay, NBA = Nijmegen Bethesda assay. | | | | | | | | | | | |

| **Table S12.** Prevalence of FVIII-specific IgA antibodies between different clinical subgroups. | | | | |
| --- | --- | --- | --- | --- |
| **Characteristics** | **IgA**  **prevalence** | **IgA positive** (n=23) | **IgA negative**  (n=765) | ***p-value*** |
| **Mean age in years (range)** |  | 44 (5-74) | 42 (0-86) | 0.680 |
| **Age categories, *n* (%)** |  |  |  | 0.859 |
| 0 – 9 years | 3.6% | 2 (8.7) | 53 (6.9) |  |
| 10 – 17 years | 1.7% | 1 (4.3) | 59 (7.7) |  |
| 18 – 35 years | 2.9% | 4 (17.4) | 132 (17.3) |  |
| 36 – 60 years | 3.5% | 12 (52.2) | 332 (43.4) |  |
| >60 years | 2.1% | 4 (17.4) | 189 (24.7) |  |
| **Hemophilia severity, *n* (%)** |  |  |  | 0.628 |
| Mild | 2.4% | 8 (34.8) | 328 (42.9) |  |
| Moderate | 4.1% | 5 (21.7) | 118 (15.4) |  |
| Severe | 3.0% | 10 (43.5) | 319 (41.7) |  |
| **Treatment regimen, *n* (%)** |  |  |  | 0.531 |
| On demand | 3.2% | 15 (65.2) | 449 (58.7) |  |
| Prophylaxis | 2.5% | 8 (34.8) | 316 (41.3) |  |
| **HCV infection^‡^, *n* (%)** |  |  |  | 0.009 |
| Never | 1.9% | 10 (43.5) | 530 (69.3) |  |
| Past | 5.2% | 13 (56.5) | 235 (30.7) |  |
| **Advanced liver fibrosis or cirrhosis in HCV-infected persons, *n* (%)** |  |  |  | 0.510 |
| No | 3.2% | 3 (23.1) | 92 (39.1) |  |
| Yes | 6.5% | 2 (15.4) | 29 (12.3) |  |
| *Missing data* |  | 8 (61.5) | 114 (48.5) |  |
| **HIV infection, *n* (%)** |  |  |  | 0.712 |
| No | 2.9% | 22 (95.7) | 742 (97.0) |  |
| Yes | 4.2% | 1 (4.3) | 23 (3.0) |  |
| **History of inhibitor development, *n* (%)** |  |  |  | 0.256 |
| No | 2.6% | 18 (78.3) | 662 (86.5) |  |
| Yes | 4.6% | 5 (21.7) | 103 (13.5) |  |
| *Abbreviations:* n = number, IgA = immunoglobulin A, HCV = hepatitis C virus, HIV = human immunodeficiency virus.  ‡ All persons with a past or current HCV infection were treated with plasma products before 1992. | | | | |

| **Table S13.** Prevalence of FVIII-specific IgG subclass, IgA, or IgM antibodies in persons with and without a history of HCV infection. | | | |
| --- | --- | --- | --- |
| **Antibody isotype or subclass** | **HCV positive, *n* (%)**  (n=248) | **HCV negative, *n* (%)**  (n=540) | ***p-value*** |
| IgG1 | 38 (15.3) | 57 (10.6) | 0.056 |
| IgG2 | 1 (0.4) | 1 (0.2) | 0.572 |
| IgG3 | 12 (4.8) | 37 (6.9) | 0.277 |
| IgG4 | 12 (4.8) | 16 (3.0) | 0.187 |
| IgA | 13 (5.2) | 10 (1.9) | 0.009 |
| IgM | 3 (1.2) | 6 (1.1) | 0.904 |
| *Abbreviations:* n = number, HCV = hepatitis C virus. | | | |

**Figures**

**Figure S1.** ELISA standard curves for IgM, IgG and IgA antibody isotype and subclasses.

**
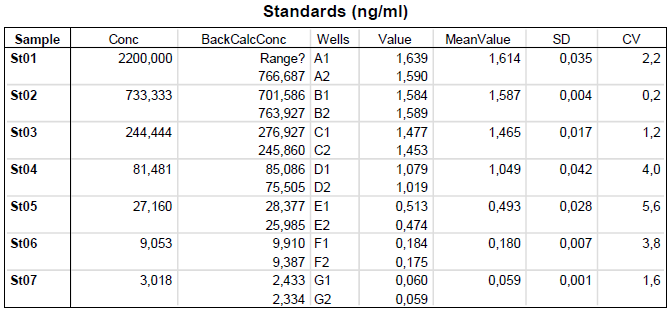

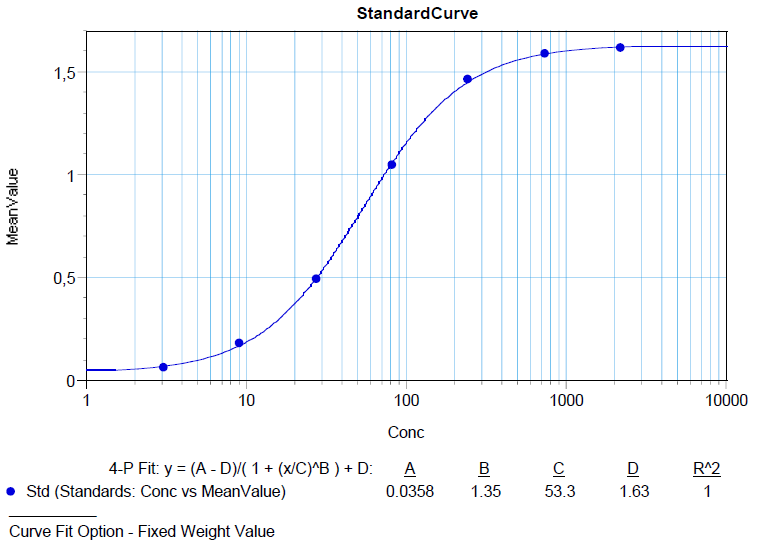
**

**(A)**


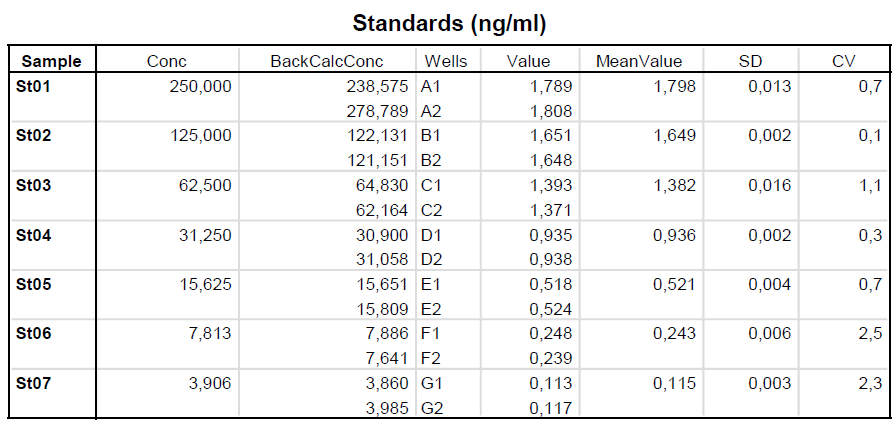

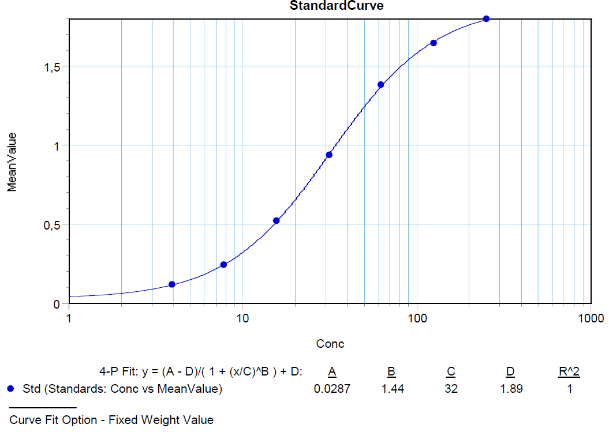

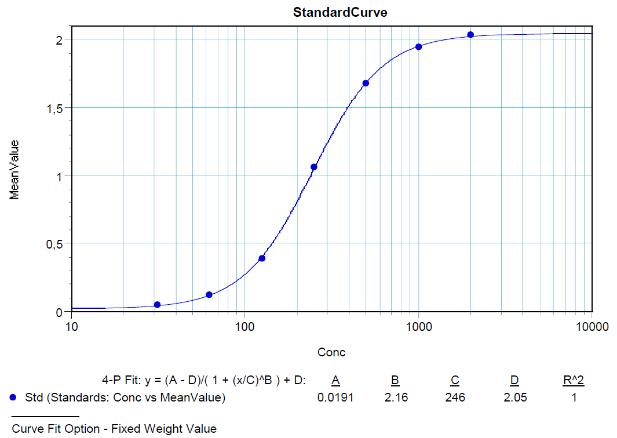

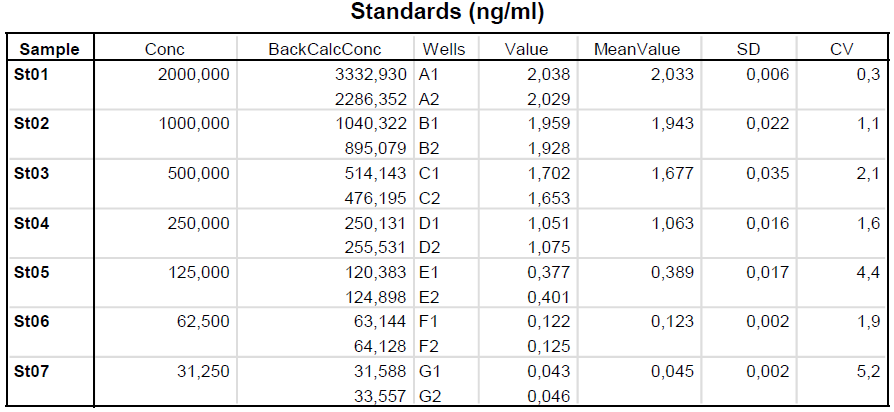

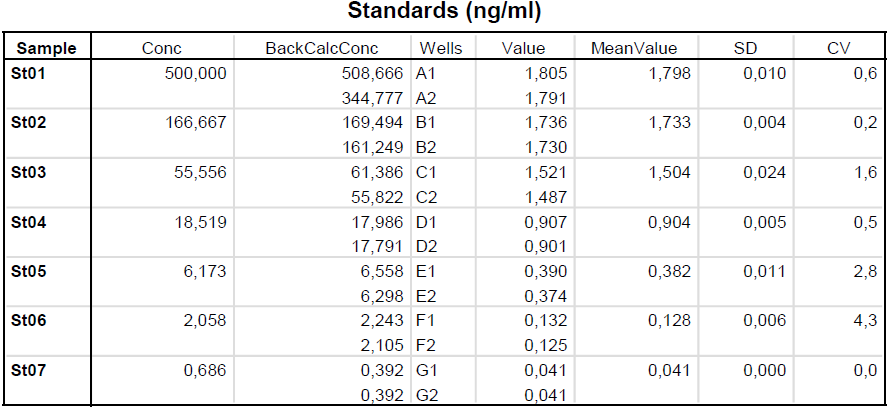

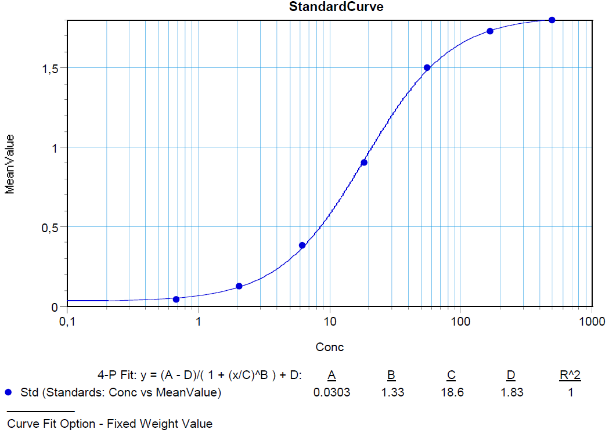
**x**

**(D)**

**(C)**

**(B)**

**
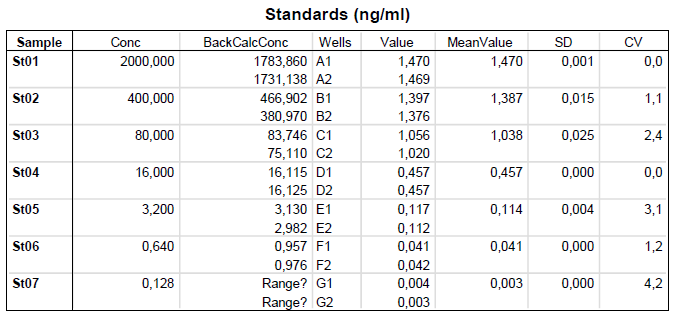

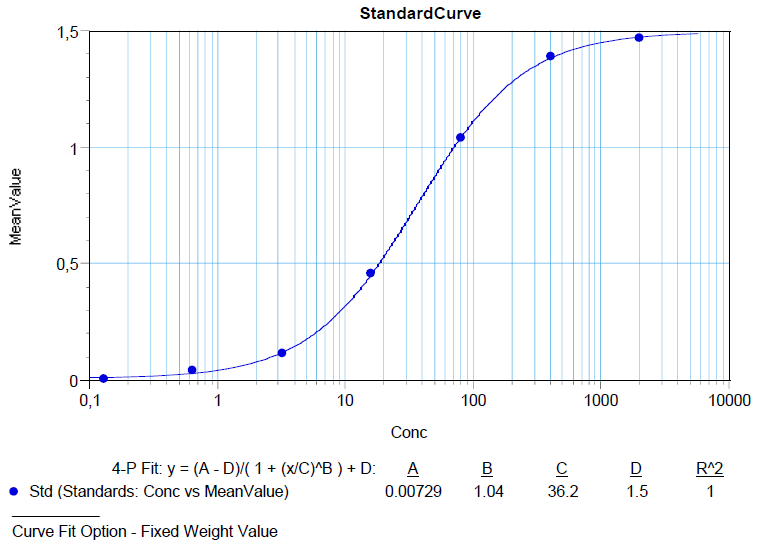
**
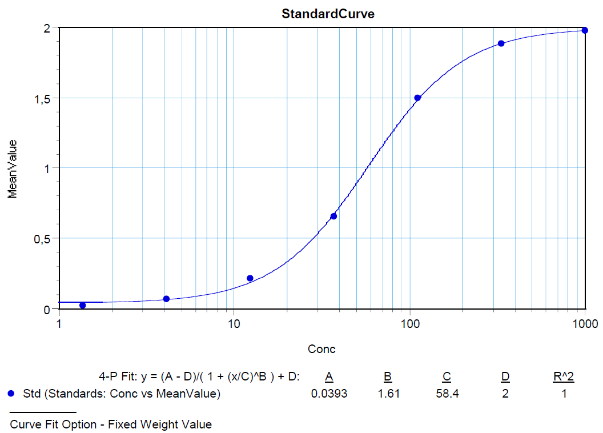

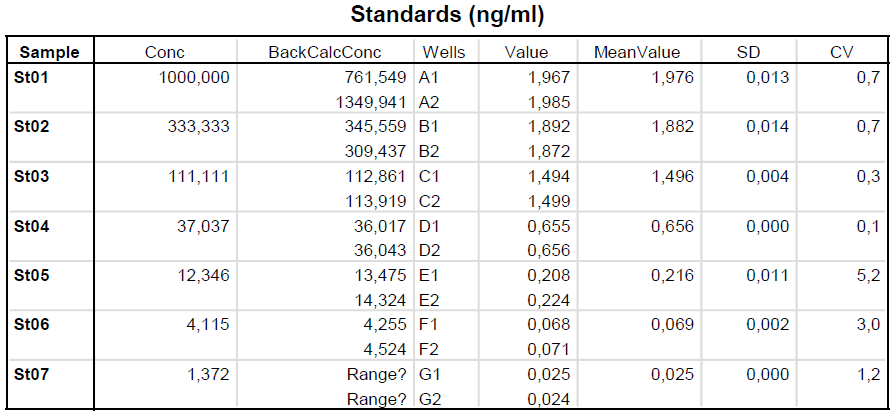


Figure **1.** Shows examples of standard curves for all Ig isotypes and IgG subclasses. **A** shows one example of the standard curve for IgM. Range is from 3.0-2200 ng/mL. This standard curve was similar for all IgM ELISAs. **B** shows one example of the standard curve for IgG1. Range is from 3.9-500 ng/mL. This standard curve was similar for all IgG1 ELISAs. **C** shows one example of the standard curve for IgG2. Range is from 31.3-2000 ng/mL. This standard curve was similar for all IgG2 ELISAs. **D** shows one example of the standard curve for IgG3. Range is from 0.7-500 ng/mL. This standard curve was similar for all IgG3 ELISAs. **E** shows one example of the standard curve for IgG4. Range is from 1.4-1000 ng/mL. This standard curve was similar for all IgG4 ELISAs. **F** shows one example of the standard curve for IgA. Range is from 0.1-2000 ng/mL. This standard curve was similar for all IgA ELISAs.

**(F)**

**(E)**

**Figure S2.** Observed signal reductions for IgG and IgM antibodies, upon competition with excess FVIII together with data obtained from the NusBA and NBA.


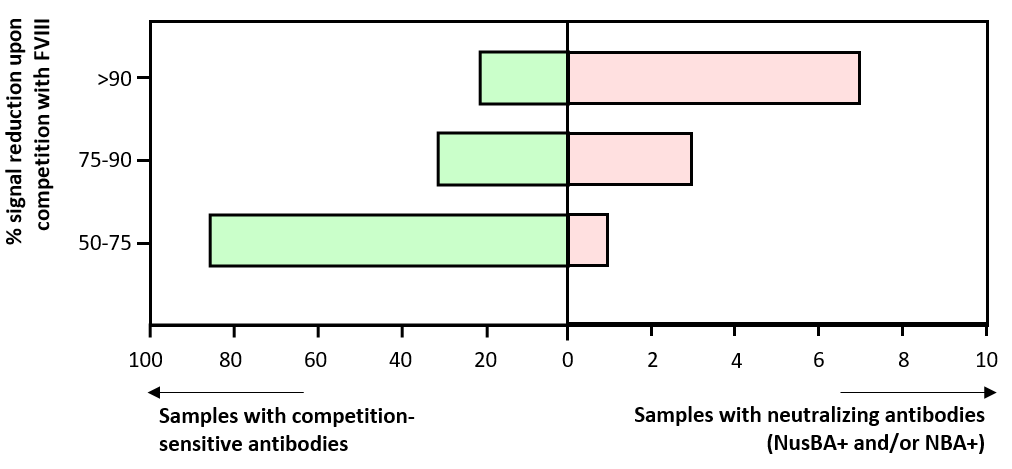


*Abbreviations:* FVIII = Factor VIII, NusBA = Nijmegen ultra-sensitive Bethesda Assay, NBA = Nijmegen Bethesda assay.

This figures presents the data of the competition ELISA together with the data obtained from the NusBA and NBA. For a subset of samples a larger reduction in signal was observed upon competition with FVIII; a 75-90% reduction of signal was observed for 55 samples whereas over 90% reduction in signal was observed for 22 samples. The samples that were positive for the NusBA or NBA were overrepresented in the 22 samples for which we observed over 90% signal reduction in the ELISA upon competition with excess FVIII.
